# The Relative Importance of Vulnerability and Efficiency in COVID-19 Contact Tracing Programmes: A Discrete Choice Experiment

**DOI:** 10.3389/ijph.2022.1604958

**Published:** 2022-07-20

**Authors:** Yi Wang, Dian Faradiba, Victor J. Del Rio Vilas, Miqdad Asaria, Yu Ting Chen, Joseph Brian Babigumira, Saudamini Vishwanath Dabak, Hwee-Lin Wee

**Affiliations:** ^1^ Saw Swee Hock School of Public Health, National University of Singapore, Singapore, Singapore; ^2^ Health Intervention and Technology Assessment Program (HITAP), Nonthaburi, Thailand; ^3^ World Health Organization - Regional Office for South-East Asia, New Delhi, India; ^4^ Global Outbreak Alert and Response Network (GOARN), Geneva, Switzerland; ^5^ London School of Economics and Political Science, London, United Kingdom; ^6^ Department of Pharmacy, Faculty of Science, National University of Singapore, Singapore, Singapore

**Keywords:** COVID-19, contact tracing, discrete choice experiment (DCE), vulnerability, efficiency

## Abstract

**Objectives:** This study aims to assess the trade-offs between vulnerability and efficiency attributes of contact tracing programmes based on preferences of COVID-19 contact tracing practitioners, researchers and other relevant stakeholders at the global level.

**Methods:** We conducted an online discrete choice experiment (DCE). Respondents were recruited globally to explore preferences according to country income level and the prevailing epidemiology of COVID-19 in the local setting. The DCE attributes represented efficiency (timeliness, completeness, number of contacts), vulnerability (vulnerable population), cooperation and privacy. A mixed-logit model and latent class analysis were used.

**Results:** The number of respondents was 181. Timeliness was the most important attribute regardless of country income level and COVID-19 epidemiological condition. Vulnerability of contacts was the second most important attribute for low-to-lower-middle-income countries and third for upper-middle-to-high income countries. When normalised against conditional relative importance of timeliness, conditional relative importance of vulnerability ranged from 0.38 to 0.42.

**Conclusion:** Vulnerability and efficiency criteria were both considered to be important attributes of contact tracing programmes. However, the relative values placed on these criteria varied significantly between epidemiological and economic context.

## Introduction

Contact tracing comprises the identification, assessment, and management of people who have been exposed to an infectious disease to prevent transmission [1]. When systematically applied, contact tracing is an essential public health tool for breaking chains of transmission and controlling infectious diseases including COVID-19 [2]. The approach to contact tracing may vary depending on the prevailing epidemiological situation and resource availability.

Contact tracing has long been used to combat public health emergencies [3]. At several points throughout the current COVID-19 pandemic, the number of cases has outpaced the public health system’s ability to identify, quarantine, and isolate all cases or potential contacts in nearly all countries, particularly in low-and-middle income countries [4]. As a result, careful planning is required to optimise contact tracing programmes in order to make the most of limited resources. During the COVID-19 pandemic, the use of contact tracing apps revolutionised this approach by allowing for timely and accurate contact identification. Nonetheless, the reliance on these apps has raised a number of concerns about privacy and equity. The most common metrics used to assess the success of contact tracing relate to efficiency, for example timeliness and completeness. Few considered the implications for equity. World Health Organization (WHO) recommended taking into account the needs of the vulnerable population when designing contact tracing programmes, which addresses the concerns around equity [1]. This is important because COVID-19’s impact has been uneven: older people, the poor, and people from historically marginalised or socially excluded groups have been disproportionately affected [5–8]. These people may not have access to healthcare, making them more susceptible to the disease’s effects. Another predominant challenge in contact tracing relates to cooperation of contacts. People are not always available for interviews or do not provide details of their close contacts, and not all contacts are reached or willing to comply with the quarantine policy [9]. A rapid review that included low, middle- and high-income countries, revealed that mistrust and/or apprehension towards the government, contact tracer, and data safety related to the use of contact tracing technology/systems all play a significant role in the lack of contact engagement. Mistrust and apprehension were also associated with information gaps. Furthermore, there was a perceived stigma associated with being a contact. People were concerned that the contact tracing system would isolate them because they would be unable to maintain routine activities, and that stigma surrounding contact tracing would lead to discrimination [10].

Given the importance of both vulnerability and efficiency considerations in conducting contact tracing, this study aimed to elicit preferences among contact tracers and other stakeholders involved in contact tracing by administering an online Discrete Choice Experiment (DCE) survey. Specifically, we focused on potential trade-offs between vulnerability and efficiency to inform public health policy design. The results of the study can be used to raise awareness of the lack of vulnerability and more broadly, equity-specific Key Performance Indicators (KPIs) and highlight the challenges of contact tracing.

## Methods

### Study Design and Participants

We conducted a DCE to elicit the preferences of global contact tracing practitioners with a focus on the trade-off between vulnerability and efficiency. DCEs have been used widely in the health economics literature to elicit individual preferences for the attributes of healthcare products and services to inform a wide range of health policy-related decisions [11, 12]. The method involves asking participants to state their preferences for hypothetical alternative options, such as goods, services, or policies. Each option is characterised by a set of attributes and levels. Preference weights for all the levels and trade-offs between different attributes can be computed. Conceptualisation of the study began in December 2020.

### Formal Discrete Choice Experiment Survey

The design of the DCE followed the good research practice guidance from the International Society of Pharmaceutical and Outcomes Research (ISPOR) [13]. Literature review was conducted first to identify the potential attributes. We then conducted a pre-pilot, in May 2021, to receive feedback on the relevance of the proposed attributes and levels for designing the DCE questionnaire through two means. First, two interactive sessions were arranged with contact tracing teams in Bangladesh and Bhutan. Second, qualitative interviews were conducted with policymakers and contact tracers to further refine the attributes and levels. The final set of six attributes and levels is presented in Table 1 with vulnerability being used to represent equity.

**TABLE 1:** Attributes and levels describing contact tracing policies (The relative importance of vulnerability and efficiency in COVID-19 contact tracing programmes: A discrete choice experiment; Global, 2021).

No	Attributes	Definition	Levels
1	Completeness	Contact identification wherein either all contacts are targeted, or contacts are prioritised based on certain criteria	Trace close contacts
			Trace all contacts
2	Timeliness	Time to reach contacts of index case (does not include follow-up)	Trace contacts within 24 h
			Trace contacts 24–48 h
			Trace contacts >48 h
3	Cooperation	Compliance with request of sharing information/engaging with contact tracer	Mandatory cooperation of contacts
			Voluntary cooperation of contacts
4	Privacy	Who has access to the contact’s personal data and how these are used	Contact tracing data used for any purpose deemed suitable by government including linking to other datasets
			Contact tracing data only used for contact tracing purposes
5	Vulnerability	The risk of having more severe symptoms due to COVID-19 infection. Vulnerable population can include elderly and people with chronic disease who are not vaccinated. In this survey, vulnerability is used as a measure of equity	Vulnerable contacts traced first
			Equal priority given to every contact regardless of vulnerability
6	Number of contacts	The number of contacts (as per local definition) that an index case comes in contact with	Contacts of cases with higher number per case traced first
			Contacts of cases given equal priority regardless of number per case

The DCE questionnaire was designed using Sawtooth version 9.11.0, and a generic (unlabeled) two-stage design was used. There were ten blocks with ten tasks in each block. For each task, in stage one, participants first selected the preferred choices from two alternative contact tracing policies. They were then asked, in stage two, to decide whether they would implement the selected contact tracing policy in real life, comparing the selected choice with an “Opt-out” option (henceforth referred to as None option). An online pilot questionnaire containing ten DCE questions was designed. To refine the questionnaire, the pilot online questionnaire was conducted in July 2021. The pilot survey was sent via email to targeted participants in Thailand from public health institutions, both at national and provincial levels; public health experts from universities; and staff working on contact tracing at WHO. Additional Information on selection of attributes and levels, pilot study and DCE design are provided in the Supplementary Material S1. The “randomiser” block function within Qualtrics was used to randomly present participants with one of ten possible sets of DCE questions. The overall study flow is summarised in the Supplementary Material S2. The pre-pilot questionnaire and the main questionnaire are presented in Supplementary Materials S3, S4, respectively.

The formal DCE survey was implemented using Qualtrics. We disseminated the survey through various channels, for example, by direct email to relevant stakeholders [e.g., Thai Department of Disease Control, Thai Department of Medical Services, HITAP country partners in the Philippines, Malaysia, Indonesia, China, Singapore, India, Japan and South Korea, Global Outbreak Alert and Response Network (GOARN), Training Program in Epidemiology and Public Health Interventions Network (TEPHINET)], and social media advertisements (e.g., Websites, LinkedIn, Twitter, Facebook and Instagram). Data collection took place from August to mid-September 2021. The target participants are individuals who had been involved in contact tracing as practitioners or experts. Search strategy for the LinkedIn survey campaign can be found in Supplementary Material S5.

### Statistical Analysis

Descriptive analyses were conducted. Left-and-right bias, where participants tend to select the choice tasks presented on one side (e.g., left-side) throughout the survey, was examined. The main analysis included a mixed-logit model and latent-class analysis to incorporate preference heterogeneity. The mixed-logit model assumes that the probability of choosing a profile from a set of alternatives (contact tracing policies) is a function of the attribute levels and a random error term that adjusts for individual-specific variations in preferences. The coefficients from the mixed-logit model for a given attribute level is referred to as the preference weights. For positive coefficients, the larger the value, the greater the preference for that particular attribute level compared to other levels. For negative coefficients, the larger the value, the lesser the preference for that particular attribute level compared to other levels. The latent-class analysis assumes that it is possible to assign respondents to unobserved classes based on their patterns of preferences. Each class has preference weights that are identical within the class yet systematically different from other classes. Akaike’s Information Criterion (AIC) and Bayesian information criterion (BIC) were used to aid model selection. Dummy coding was used in the analysis with the reference attribute levels having preference weight 0.

Country income level, as defined by the World Bank Group classifications [14], and self-reported local COVID-19 transmission conditions were used as control variables in the analysis. Due to the relatively small sample size, participants from lower-middle-income countries (LMICs) and low-income countries (LICs), and participants from high-income countries (HICs) and upper-middle-income countries (UMICs) were combined to form the low-to-lower-middle income countries (LLMIC) and the upper-middle-to-high income countries (UMHIC), respectively. Additionally, due to the small sample size, participants from countries with sporadic cases and clusters were combined into a single group (sporadic cases/clusters). These control variables were used to capture the systematic (as opposed to random) preference heterogeneity across four settings: LLMIC with sporadic cases/clusters (reference setting), LLMIC with community transmission, UMHIC with sporadic cases/clusters and UMHIC with community transmission.

In the latent-class analysis, the control variables affect the probabilities of the participants being assigned to different unobserved classes. In the mixed-logit model, if the preference weights ( $\beta_{UMHIC}$ and $\beta_{com}$ ) for the interaction terms between the attributes ( $x_{ij}$ ) and settings ( $I_{UMHIC,i}$ and $I_{com,i}$ ), as shown in Eq. 2, are statistically significant, then systematic preference heterogeneity is said to be present. The detailed specifications of mixed-logit model with systematic preference heterogeneity are as follows:

The random utility $U_{ij}$ of individual *i*, for a contact tracing policy *j*, is

$$U_{ij}=\beta_{i}*x_{ij}+\epsilon_{ij}$$ (1)

$$\beta_{i}=\beta +\beta_{UMHIC}*I_{UMHIC,i}+\beta_{com}*I_{com,i}+\eta_{i}$$ (2)

where $x_{ij}$ is a vector of the observed contact tracing policy attributes; $\epsilon_{ij}$ is the idiosyncratic error term following type 1 extreme value distribution. $\beta_{i}$ is a vector measuring the preference of individual i for levels that taken by attributes $x_{ij}$ , with: β : the mean preference weights for people in the reference setting (LLMIC and sporadic cases/clusters), $I_{UMHIC,i}$ : indicator function with value 1 if individual i is from UMHIC and 0 if individual *i* is from LLMIC, $I_{com,i}$ : indicator function with value 1 if individual i is from a country with community transmission and 0 otherwise, $\beta_{UMHIC}$ : additional preference weight for people from UMHIC, measuring the systematic preference heterogeneity, $\beta_{com}$ : additional preference weight for people from countries with community transmission, measuring the systematic preference heterogeneity, $\eta_{i}$ : follows multivariate normal distribution, adjusting individual-specific variations in preference (the random preference heterogeneity). Analyses considering one control variable at a time were conducted first. The interaction between each control variable and all the levels were subsequently examined. Considering the sample size, only statistically significant interaction terms from each model were selected and entered the final model. We also presented the results using standard mixed-logit model without systematic preference heterogeneity, i.e., $\beta_{UMHIC}=0$ and $\beta_{com}=0$ .

Conditional relative importance for a given attribute, defined as the difference between the highest preference weight of the attribute level and the lowest preference weight of the attribute level, was reported. A higher conditional relative importance indicates the attribute is more important in designing the contact tracing policy. The uptake probabilities of the most preferred and least preferred contact tracing policies were examined to evaluate their implementability. To define a contact tracing policy, one and only one level needs to be selected for each attribute. The most (least) preferred contact tracing policy is the policy with the highest (lowest) sum of the preference weights of the selected levels. First, we presented the uptake probabilities for the most preferred contact tracing policy and the least preferred contact tracing policy, under the condition that these are the only two options and one of them must be chosen. This corresponds to stage one in the DCE questions, choosing a preferred contact tracing policy between two alternative policies. Second, we presented the uptake probability for each of the most preferred and least preferred policy, under the condition that the given policy is the only option and either that policy is implemented, or no contact tracing programme will be implemented. This corresponds to stage two in the DCE questions.

All statistical analyses were carried out using R (Version: 4.0.3) [15].

## Results

Most participants (N = 114 out of 181) were from LMICs (Table 2), with 112 participants indicating that they were experiencing community transmission and a third were directly involved in contact tracing. Number of participants per block was similar, ranging from 15 to 21. Median time to complete the survey was 16 minutes. About 45% of respondents received fixed payments (Supplementary Table S6.2). The mixed-logit model returned a smaller AIC and BIC compared to the latent-class analysis. Hence, we focused on discussing the results from the mixed-logit model in the main text. Due to the word limit, results from latent-class analysis are presented in Supplementary Table S6.3.

**TABLE 2:** Summary statistics (The relative importance of vulnerability and efficiency in COVID-19 contact tracing programmes: A discrete choice experiment; Global, 2021).

Characteristics	Study sample (*N* = 181)	
	N	%
Country income level		
High income	20	11.04
Upper middle income	42	23.20
Lower middle income	114	62.98
Low income	5	2.76
Region^a^		
Europe and central Asia	10	5.52
East Asia and Pacific	51	28.17
South Asia	91	50.27
North America	4	2.20
Latin America and Caribbean	3	1.65
Sub-Saharan Africa	22	12.15
Epidemiology condition		
Sporadic case	36	19.88
Cluster case	33	18.23
Community transmission	112	61.87
Education level		
Bachelor/Masters/PhD	159	87.84
High school diploma or equivalent	5	2.76
Other	12	6.62
Prefer not to say	5	2.76
Role in contact tracing		
Academic/expert in contact tracing	35	19.33
Contact tracer	23	12.70
Contact tracer manager/supervisor	58	32.04
Policy makers	16	8.83
Others	25	13.81
Prefer not to say	24	13.25

a Details on response by country can be found in Supplementary Table S6.1.

Preference weights from the mixed-logit model are presented in Table 3, which focuses on comparing the levels within each attribute. Table 3, Column A shows the results from the standard mixed-logit model considering random preference heterogeneity only, i.e., no interaction terms between settings and attributes. The results show the preferences across the entire sample. There was no left-and-right bias as indicated by the insignificant coefficient (variable Right, coefficient = −0.10, *p*-value = 0.100). A positive coefficient of a level indicates that participants attach higher preference weight to that level compared to the reference level. Overall, participants preferred tracing contacts faster (coefficient = 0.67, *p*-value < 0.001; coefficient = 0.60, *p*-value < 0.001), a mandatory contact tracing programme (coefficient = 0.18, *p*-value = 0.039), prioritizing vulnerable population (coefficient = 0.29, *p*-value < 0.001), and tracing people with high number of contacts first (coefficient = 0.19, *p*-value = 0.010). When random preference heterogeneity at the individual level was examined, all parameters were significant.

**TABLE 3:** Preference weights from mixed-logit model (Reference setting for Column B: LLMIC with sporadic cases/clusters) (The relative importance of vulnerability and efficiency in COVID-19 contact tracing programmes: A discrete choice experiment; Global, 2021).

Attributes and levels	Column A		Column B	
	Settings not controlled		Settings controlled	
	Coefficient	*p*-value	Coefficient	*p*-value
Right	−0.10	0.100	−0.11	0.095
None option	−2.43	<0.001	−3.51	<0.001
None option # community transmission	—	—	0.16	0.712
None option # UMHIC	—	—	2.07	<0.001
Completeness				
Trace close contacts	0.15	0.097	−0.10	0.463
Trace close contacts # community transmission	—	—	0.39	0.030
Trace all contacts	Reference	Reference	Reference	Reference
Timeliness				
Less than 24 h	0.67	<0.001	0.71	<0.001
24–48 h	0.60	<0.001	0.50	<0.001
24–48 h # UMHIC	—	—	0.28	0.068
>48 h	Reference	Reference	Reference	Reference
Cooperation				
Mandatory cooperation of contacts	0.18	0.039	0.20	0.025
Voluntary cooperation of contacts	Reference	Reference	Reference	Reference
Privacy				
Contact tracing data only used for contact tracing purpose	0.06	0.472	0.09	0.339
Contact tracing data used for any purpose deemed suitable by government including linking to other database	Reference	Reference	Reference	Reference
Vulnerability				
Trace vulnerable population first	0.29	<0.001	0.30	<0.001
Equal priority given to every person regardless of vulnerability	Reference	Reference	Reference	Reference
Number of contacts				
Trace index case with high number of contacts first	0.19	0.010	0.07	0.469
Trace index cases with high number of contacts first # UMHIC	—	—	0.37	0.015
Equal priority given to every person regardless of number of contacts	Reference	Reference	Reference	Reference
Random preference heterogeneity at individual level (measures the individual-specific variations in preference)				
None option	2.89	<0.001	3.02	<0.001
Completeness, close contact	0.74	<0.001	0.67	<0.001
Timeliness, <24 h	0.41	0.004	0.40	0.005
Timeliness, 24–48 h	0.22	0.156	0.30	0.028
Cooperation, mandatory	0.83	<0.001	0.74	<0.001
Privacy, contact tracing data only used for contact tracing purpose	0.84	<0.001	0.84	<0.001
Vulnerability, trace vulnerable population first	0.43	0.001	0.53	<0.001
Number of contacts, trace index case with high number of contacts first	0.41	0.001	0.42	<0.001
Model fit				
AIC	3,228.0		3,210.2	
BIC	3,333.3		3,346.0	

# denotes interaction term e.g., Trace close contacts # community case.

Reference category is defined as having a coefficient with value of zero, indicated with Reference in the table.

Column A shows the results without controlling settings.

Column B shows the results with settings being controlled.

To interpret the coefficient, None option # UMHIC = 2.07 means that, for None option, the preference weights for participants from UMHIC were 2.07 higher than the preference weights for participants from LLMIC. Given that the preference weights for participants from LLMIC was −3.51, the preference weights for participants from UMHIC was −1.44.

AIC, Akaike information criterion; BIC, Bayesian information criterion; UMHIC, upper-middle-to-high income countries; LLMIC, low-to-lower-middle income countries.

Table 3, Column B shows the results considering both systematic preference heterogeneity and random preference heterogeneity. The results should be interpreted with the setting of LLMIC and sporadic or cluster as the reference setting. There was no left-and-right bias as indicated by the insignificant coefficient (variable Right, coefficient = −0.11, *p*-value = 0.095). There were systematic preference heterogeneities (as reflected by the statistically significant interaction term) for none option, and three of the attributes: completeness, timeliness, and number of contacts. A statistically significant positive interaction term (e.g., trace index cases with high number of contacts first # UMHIC, coefficient = 0.37, *p*-value = 0.015) indicates that UMHIC participants have significantly higher preference weights than LLMIC participants. In this case, the preference weight for tracing index case with high number of contacts first for UHMIC participants is 0.07 + 0.37 = 0.44. That is to say, the difference between the two preference weights is the coefficient of the interaction term (0.44–0.07 = 0.37). To give another example, participants from UMHIC have a higher preference (interaction term +2.07) for none option, or to NOT implement (negative preference weight, −3.51 + 2.07 = −1.44) any given contact tracing policy compared to participants from LLMIC.

**FIGURE 1:**
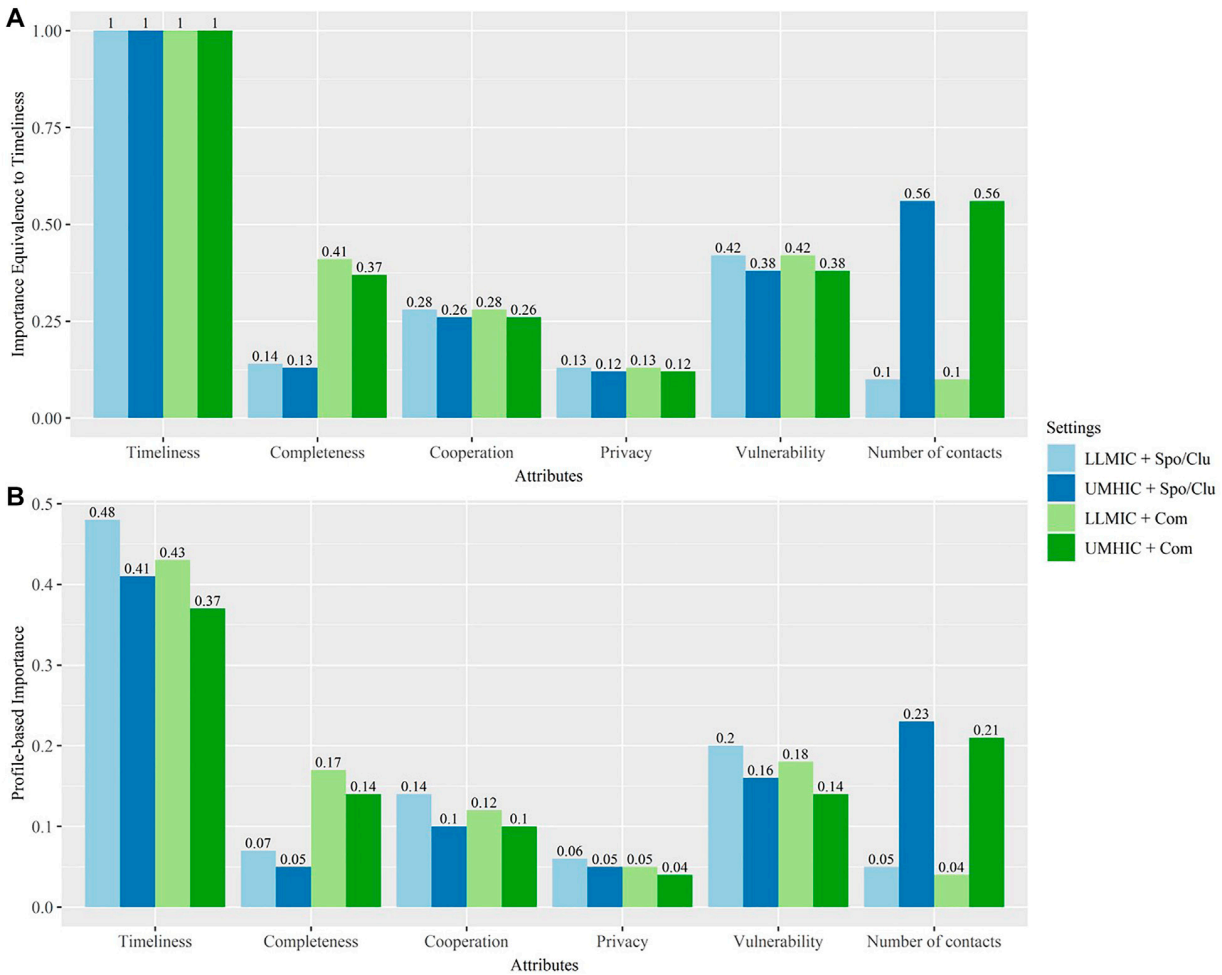
Conditional Relative Importance (The relative importance of vulnerability and efficiency in COVID-19 contact tracing programmes: A discrete choice experiment; Global, 2021). Note: This figure illustrates the importance of various attributes and facilitates between-attributes comparisons. In attribute-based normalisation [Panel **(A)**], we show how important each attribute is, relative to timeliness as timeliness is the attribute with the highest conditional relative importance [i.e., conditional relative importance of all the attributes were normalised to conditional relative importance of timeliness (*y*-axis)]. In profile-based normalisation [Panel **(B)**], the profile-based importance within each setting sums up to one. This provides a better picture of the relative importance of various attributes in the contact tracing policy design within each setting. UMHIC, upper-middle-to-high income countries; LLMIC, low-to-lower-middle income countries; Spo/Clu, sporadic cases and cluster cases; Com, community transmission.

As the results from Table 3, Column B shows the existence of systematic preference heterogeneity, we focused on exploring the conditional relative importance and uptake probability across different settings. From Figure 1A, we observed that the vulnerability attribute is approximately 38%–42% as important as timeliness. The ranking of attributes relative to timeliness depends on the setting. For example, the second most important attribute for LLMIC participants is vulnerability while that for UMHIC participants is number of contacts.

From Figure 1B, we are able to understand the relative importance of all attributes within a single setting. For example, within the LLMIC and sporadic cases/clusters setting, timeliness is the most important attribute (0.48) followed by vulnerability (0.2), cooperation (0.14), completeness (0.07), privacy (0.06), and number of contacts (0.05). Statistical tests were conducted using the Krinsky and Robb method [16]. Timeliness is statistically more important than the remaining attributes for all the settings. For UMHIC, number of contacts is statistically more important than privacy.

Table 4 shows the most preferred contact tracing policies and the least preferred contact tracing policies across the four settings. Assuming that one of two policy options must be chosen, the uptake probability for the most preferred contact tracing policy was 3–4 times that for the least preferred contact tracing policy regardless of setting. Assuming that the policy being evaluated is the only option, the uptake probability for the least preferred contact tracing policy varies from 64.3% to 83.0% across the four settings.

**TABLE 4:** Most Preferred and Least Preferred Contact Tracing Policy (The relative importance of vulnerability and efficiency in COVID-19 contact tracing programmes: A discrete choice experiment; Global, 2021).

Attributes	Settings							
	Sporadic cases/cluster, low-to-lower-middle income		Sporadic cases/cluster, upper-middle-to-high income		Community, low-to-lower-middle income		Community, upper-middle-to-high income	
	Most preferred	Least preferred	Most preferred	Least preferred	Most preferred	Least preferred	Most preferred	Least preferred
Completeness	Trace all*	Trace close contacts*	Trace all*	Trace close contacts*	Trace close contacts	Trace all	Trace close contacts	Trace all
Timeliness	<24 h	>48 h	24–48 h	>48 h	<24 h	>48 h	24–48 h	>48 h
Cooperation	Mandatory	Voluntary	Mandatory	Voluntary	Mandatory	Voluntary	Mandatory	Voluntary
Privacy	Data only for CT purpose*	Data for CT as well as others*	Data only for CT purpose*	Data for CT as well as others*	Data only for CT purpose*	Data for CT as well as others*	Data only for CT purpose*	Data for CT as well as others*
Vulnerability	Vulnerable first	Equal priority regardless of vulnerability	Vulnerable first	Equal priority regardless of vulnerability	Vulnerable first	Equal priority regardless of vulnerability	Vulnerable first	Equal priority regardless of vulnerability
Number of contacts	Contacts of cases with higher number per case traced first*	Contacts of cases given equal priority regardless of number per case*	Contacts of cases with higher number per case traced first	Contacts of cases given equal priority regardless of number per case	Contacts of cases with higher number per case traced first*	Contacts of cases given equal priority regardless of number per case*	Contacts of cases with higher number per case traced first	Contacts of cases given equal priority regardless of number per case
Uptake probability if one of two policy options must be chosen (Stage 1)	73.8%	26.2%	79.8%	20.2%	76.4%	23.6%	82.1%	17.9%
Uptake probability if the evaluated policy is the only option (Stage 2)	90.2%	83.0%	80.6%	64.7%	90.4%	83.0%	81.2%	64.3%

*Not significant coefficient.

Notes: For the levels indicated with *, statistically, participants considered the levels the same within the corresponding attributes.

CT: contact tracing.

Among the most preferred policies, we observed that the levels were similar for most attributes except for completeness and timeliness. The level of these attributes depended on either the country income level or the local epidemiological condition. Both LLMIC and UMHIC participants preferred to trace all when the local epidemiological condition is sporadic cases/clusters compared to community transmission [17]. However, regardless of the local epidemiological condition, LLMIC participants preferred to complete contact tracing within 24 hours while UMHIC participants preferred to complete tracing between 24 and 48 hours. There is greater similarity across the settings for the least preferred policies. The only attribute that differed across the settings was completeness. Both LLMIC and UMHIC participants least preferred to trace all when the local epidemiological condition is community transmission compared to sporadic cases or clusters.

## Discussion

To the best of our knowledge, this is the first study that explored the trade-off between vulnerability and efficiency in contact tracing using a DCE. Vulnerability, a health equity stratifier was used as an indicator for equity in this study [1], which an attribute highlighted by WHO in its guidance on contact tracing. None of the few DCE studies involving contact tracing have examined the equity aspect of contact tracing. For example, Jonker et al. [18] examined what type of warning Dutch COVID-19 application users would like to receive, if app users would be offered testing, and whether they reported to health authorities if they were found to be within close proximity of a COVID-19 case. Frimpong and Helleringer [19] focused on the accuracy and sensitivity of COVID-19 app notifications, how app privacy would be managed, and whether app users would be financially incentivised within the United States. Similarly, a British study by Wiertz et al. [20] also looked at how COVID-19 app data would be handled, and who had the responsibility of oversight; they also examined the type of warning alerts and if reporting of COVID-19 test result would be compulsory like Jonker et al. [18]. The attributes used in these three DCE studies reflect the priorities of Western countries on privacy and efficiency in conducting COVID-19 contact tracing. Yet among LMICs, equity barriers of contact tracing abound: a study of 13 country case studies by Shadmi et al. [21] highlighted how intra-country regional inequalities, racial inequalities and lack of equal access to healthcare can lead to the pandemic inflicting disproportionate harm on the global poor. While some people may be concerned about using DCE in LLMIC setting [22], we found that this may not be the case for our study. For instance, participants did not take shortcut when completing the DCE as evidenced by the lack of left-right bias.

Our study shows the trade-off between vulnerability and efficiency attributes which varies by setting as demonstrated by the presence of systematic preference heterogeneity. Even within a setting, individuals’ preferences vary as suggested by the existence of random preference heterogeneity. Estimation results can be biased if preference heterogeneity is not accounted for in modelling [23]. While efficiency was given more weight than vulnerability, vulnerability was considered among the most important attributes for contact tracing, ranking second or third across four settings among the six attributes.

Our original objective was to understand the trade-offs between equity and efficiency in designing contact racing programme. We considered several attributes measuring equity in the pre-pilot and pilot studies, e.g., location of contacts, proximity to healthcare facility, etc., and dropped them eventually based on feedback from participants. For example, we were informed that location of contacts which was supposed to differentiate contact tracing priorities in urban versus rural setting was not relevant in Sub-Saharan Africa. Vulnerability was chosen to represent equity based on the comments received from pilot and pre-pilot, which could reflect that people care about the larger health impact of the disease on the vulnerable population. One policy implication of the study results is that contact tracing programmes should be designed to prioritising vulnerable populations. However, this does not mean that contact tracing programmes should always trace the vulnerable population first. Rather, this implies that the design of the contact tracing programme should make allowance to forgo certain amount of efficiency in exchange for equity. Our results can help identify the amount of efficiency that the stakeholders are willing to sacrifice. For example, if prioritising the vulnerable population leads to too much extra time to complete tracing one contact, from less than 24 hours to more than 48 hours, in this case, the policymakers need to carefully consider whether to prioritise the vulnerable population. The coefficients of the attributes can be used as weights to inform compensation decisions and support systems for resource allocation by contact tracing managers. However, additional issues may need to be considered in reality such as cognitive bias. There could also be additional utility loss from forgoing opportunities, e.g., loss aversion.

In this study, privacy was not found to be important and mandatory cooperation was preferred over voluntary cooperation to supply information for contact tracing. This may have been on account of the audience of the study, who were primarily contact tracers or practitioners. Jonker et al. [18] examined preferences for a contact tracing app among users and found that a secure and privacy-respecting contact tracing app was preferred over no contact tracing app at all in the Netherlands. Hence, there may be a difference in preferences between contact tracers/practitioners and the general public. Therefore, the design of contact tracing policies should consider the preferences of different groups.

An important strength of our paper is that we analysed our data by four settings defined by country income level (LLMIC vs. UMHIC) and COVID-19 transmission conditions (sporadic cases or clusters vs. community transmission). We believe that no other DCE study examined preferences for contact tracing policy by these settings. It is clear that no one policy fits all situations and our findings provided insights into what would be preferred by our study participants under various settings. The differences in preferences could be explained by many factors, such as resource availability, feasibility of completing the task, urgency of tracing the contact as soon as possible and the cost of missing a contact [24]. We observed that UMHIC participants prefer to trace index cases with high number of contacts than to trace all index cases while participants in LLMIC do not have a preference. From the efficiency point of view, it makes sense to trace index cases with high number of contacts first. However, in the LLMIC setting, perhaps, it is not easy to determine the number of contacts as there may be low adoption rate of smartphones, limited access to WiFi, or lack of tools such as digital contact tracing app [25–27]. Other factors such as relative housing density and occupation type could be correlated with number of contacts and affect people’s preferences. However, these factors are expected to affect both LLMIC and UMHIC [28, 29]. From the equity point of view, perhaps there should be no differentiation in terms of whom to trace first and all contacts ought to be treated as equally important. Hence, this represents a trade-off between equity and efficiency and further engagement with the contact tracing teams will be required to better understand their preferences.

Our observation that UMHIC participants are less likely to implement any of the contact tracing policies, suggests that perhaps our attributes and levels did not fully capture their preferences, given that our pre-pilot and pilot were conducted among LLMIC participants. Nonetheless, given the scenario that if the given policy is the only option, we observed that about 64% of UMHIC participants will implement even the least preferred policy and about 81% of UMHIC participants will implement the most preferred policy. It is noteworthy that while we observed differences between UMHIC and LLMIC participants, these are limited to one to two of the six attributes only.

There are a few limitations to our study. We collected the data through an online survey. Hence, we were unable to identify the total number of people that received the invitation, the participation rate and factors that were correlated with participation decision. There could be selection bias due to participation decisions, which we were unable to examine directly. It is common for DCEs to include a cost attribute to calculate the willingness to pay for a particular policy option. To that effect, providing monetary incentives for contact tracers was an attribute that the study team considered but was eventually not included. This is due to the challenges of standardising the cost attribute across multiple countries, given variation in country income level and varying practices of reimbursing contact tracers.

An equity issue that we have not considered is whether people who are quarantined should be compensated. For example, migrant workers who are typically daily wage earners as well as others such as street vendors, construction workers, taxi drivers, may require compensation to support their families if they are quarantined. Daily wage earners in general may be less cooperative or less able to comply with contact tracing activities to avoid financial losses [30–35] and this may therefore compromise the efficiency of contact tracing. Hence, providing financial support during quarantine and for regular testing may need to be considered by governments. This is already provided by several UMHIC countries including the UK and Australia [36, 37]. In addition, the sample size (181 respondents) limited our ability to conduct in-depth sub-group analyses or draw conclusions that are generalisable. The response rate was low possibly because priority is given to contact tracing duties.

Suggestions for further research include identifying additional equity-related attributes for contact tracing and a better understanding of how equity considerations may be incorporated into current contact tracing protocols. As contact tracing is a localised policy, national or sub-national level DCEs should be conducted to inform local policies. Further, as this study only focused on the practitioners and experts, future studies should examine the preferences from diverse groups, such as those who are being traced. Finally, the study highlights that while considered important, equity-related measures are limited and there is scope to explore this issue further.

### Conclusion

Our study showed that efficiency in terms of timeliness is the most important attribute in contact tracing with equity coming in a close second or third. There are differences in preferences according to country income level as well as local epidemiological conditions. Since contact tracing is an important policy measure during a pandemic, a better understanding of how to design contact tracing policies across different settings will remain relevant even with the advent of vaccines.
