# Supplementary material for: The Relative Importance of Vulnerability and Efficiency in COVID-19 Contact Tracing Programmes: A Discrete Choice Experiment
Source: Int J Public Health. 2022 Jul 20;67:1604958. doi: 10.3389/ijph.2022.1604958 (PMC9346065; doi:10.3389/ijph.2022.1604958)
Supplement: Supplementary file 1 [file DataSheet1.docx]

**Original Article:**

**The relative importance of vulnerability and efficiency in COVID-19 contact tracing programmes: a discrete choice experiment**

**Supplements**

[S1. Selection of attributes and levels 3](#_Toc105404636)

[S2 Supplementary figure 5](#_Toc105404637)

[S3. Pre-pilot (interview) guide 7](#_Toc105404638)

[S4. Main survey questionnaire 12](#_Toc105404639)

[S5. Full DCE survey question 15](#_Toc105404640)

[S6. LinkedIn campaign strategy 19](#_Toc105404641)

[S6. Supplementary tables 19](#_Toc105404642)

[S7. Summary of literature review 25](#_Toc105404643)

[**References** 51](#_Toc105404644)

## S1. Selection of attributes and levels

We conducted a literature review to identify the initial set of attributes and the corresponding levels. Two search approaches were used to consider both the efficiency and equity aspects of contact tracing. First, a literature search was performed to find DCE studies focused on contact tracing for COVID-19. A second literature search was conducted to identify attributes related to contact tracing in general, beyond COVID-19. This second review was used to identify the initial list of attributes that were considered for the study. A list of 10 attributes, which were initially proposed, can be found in Table S2.1.

We conducted a pre-pilot to receive feedback on the relevance of the proposed attributes and levels for designing the DCE questionnaire through two means. First, two interactive sessions were arranged with contact tracing teams in Bangladesh (35 participants) and Bhutan (6 participants). They had various roles in contact tracing such as contact tracer, contact tracer supervisor, case investigator, data manager, researcher, and contact tracing technical support. Participants were asked to rank the attributes and select the top attributes they considered important. They were also asked whether the levels were appropriate. Open-ended questions were used to elicit general comments about the study.

Second, qualitative interviews were conducted with policymakers and contact tracers to further refine the attributes and levels. Relevant stakeholders were identified from public health institutions at the national and provincial levels, as well as public health experts from universities in Thailand. The invitation to participate in the pre-pilot was sent via email. Five potential participants agreed to participate and were interviewed out of a total of sixteen. Reimbursement was given as compensation for their time. One-hour interviews consisted of four sections including a discussion about the background information to understand how the COVID-19 contact tracing was being conducted, going through attributes and levels one by one and discussing their importance, ranking the attributes, and an open discussion on other potential issues such as equity and accuracy of contact tracing. The interviews were audio-recorded and transcribed.

**Table S2.1 Proposed attributes and levels (The relative importance of vulnerability and efficiency in COVID-19 contact tracing programmes: a discrete choice experiment; Global; 2021)**

| No | Attributes | Levels |
| --- | --- | --- |
| 1 | Contacts traced per tracer per day | 0-5 |
|  |  | 5-10 |
|  |  | >10 |
| 2 | Completeness | Trace close contacts |
|  |  | Trace all contacts |
| 3 | Timeliness | Trace contacts within 24 hours |
|  |  | Trace contacts 24-48 hours |
|  |  | Trace contacts > 48 hours |
| 4 | Setting | Trace urban as priority |
|  |  | Trace rural as priority |
|  |  | Trace all regardless settings |
| 5 | Social Economic Status (SES) | Trace low SES as priority |
|  |  | Trace all regardless SES |
| 6 | Incentive (to tracer) per day | $0 |
|  |  | $1 |
|  |  | $5 |
|  |  | $10 |
|  |  | $20 |
| 7 | Epidemiology | Target sporadic case contacts |
|  |  | Target cluster contacts |
|  |  | Target community contacts |
| 8 | Acceptability | Contacts engage with contact tracing voluntarily |
|  |  | Contacts engage with contact tracing only if mandatory |
| 9 | Accuracy (of tests) | Test accurate |
|  |  | 5% false positive rate |
|  |  | 10% false positive rate |
| 10 | Privacy | Mandatory reporting of contact to authorities |
|  |  | Reporting of contact to authorities only with consent |
|  |  | Reporting of contact to authorities voluntarily |

Pilot study:

An online pilot questionnaire containing ten DCE questions was designed. We included demographic questions at the beginning and evaluation questions at the end to assess the appropriateness of the questionnaire. Three scenarios representing different epidemiological contexts, which were defined based on WHO standard definition for COVID-19 transmission scenarios (sporadic cases, cluster of cases, and community transmission),[1] were tested; participants were randomly assigned to consider these contexts. The pilot online questionnaire was sent via email to 23 targeted participants in Thailand from public health institutions, both at national and provincial levels; public health experts from universities; and staff working on contact tracing at WHO. Thirteen participants completed the questionnaire with eight, three and two responses for scenario one (sporadic cases), scenario two (cluster of cases), and scenario three (community transmission), respectively. Reimbursement was not given to survey respondents due logistical challenges. We refined the DCE questionnaire based on the comments from the pilot study.

DCE Design:

The design of the DCE followed the good research practice guidance from the International Society of Pharmaceutical and Outcomes Research (ISPOR).[2] The final set of six attributes and levels is presented in Table 1. Information on selection of attributes and levels and pilot study are provided in the Appendix. The DCE questionnaire was designed using Sawtooth version 9.11.0, and a generic (unlabeled) two-stage design was used. For each task, in stage one, participants first selected the preferred choices from two alternative contact tracing policies. They were then asked, in stage two, to decide whether they would implement the selected contact tracing policy in real life, comparing the selected choice with an “Opt-out” option (henceforth referred to as None option). The latter allows us to predict uptake of each contact tracing policy. The questionnaire was designed using the Balanced Overlap option provided by Sawtooth.[3] Only main effects were considered. Ten blocks were used, with eight random tasks and two fixed tasks per block. Fixed tasks are seen by all participants whereas random tasks are randomly assigned with each task being seen by a subset of participants. A simulation exercise was conducted to examine the coverage matrix of the design and test the sample size required. An example of DCE choice task is presented in Figure below.

**Figure S1.1 An example of DCE choice task The relative importance of vulnerability and efficiency in COVID-19 contact tracing programmes: a discrete choice experiment; Global; 2021)**


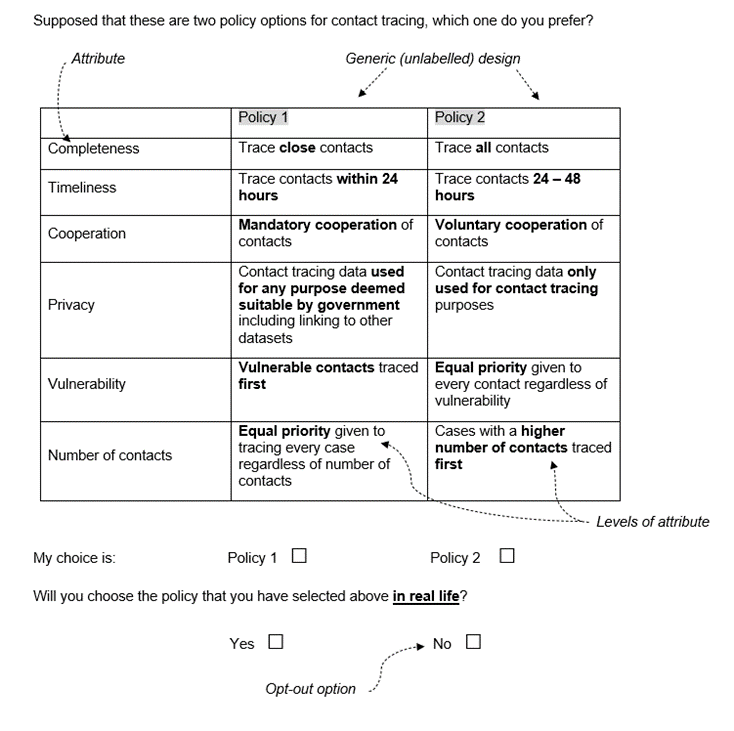


Other information collected in the questionnaire included participant’s education level, country of residence and role in contact tracing. Questions on receipt of monetary incentives and incentive payment modes were also included. Acting on feedback from participants in the pilot study, we allowed participants to select their local COVID-19 transmission conditions from sporadic cases, cluster of cases, and community transmission before the DCE survey commenced. Participants then completed the DCE and chose their preferred contact tracing policies considering their local COVID-19 transmission conditions.

## S2 Supplementary figure

**Figure S2.1 Overall study flow (The relative importance of vulnerability and efficiency in COVID-19 contact tracing programmes: a discrete choice experiment; 2021)**


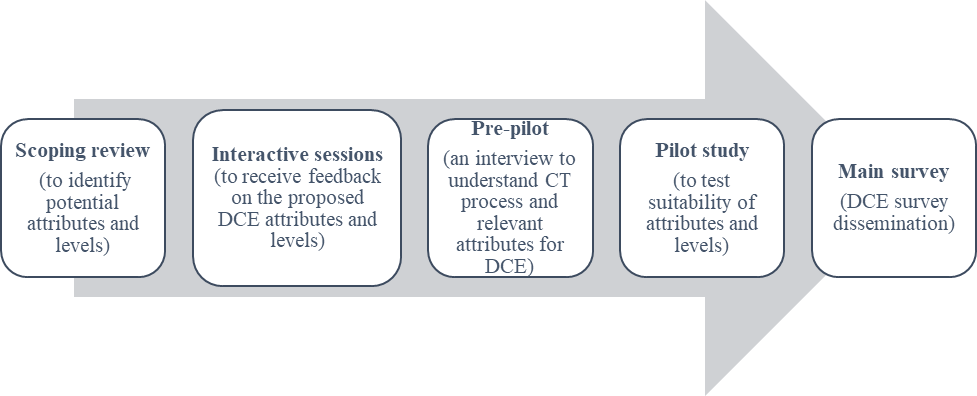


**Figure S2.2 Study timeline (The relative importance of vulnerability and efficiency in COVID-19 contact tracing programmes: a discrete choice experiment; 2021**


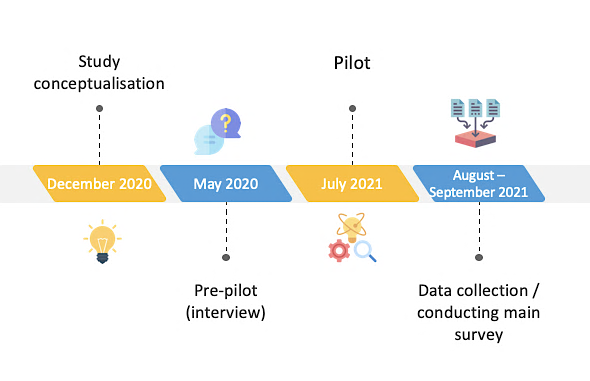


## S3. Pre-pilot (interview) guide

Main Study Objectives:

1. To review the scope and challenges of conducting contact tracing (CT) during COVID-19
2. To elicit contact tracing preferences among relevant stakeholders in the World Health Organization Southeast Asia Regional Office (WHO SEARO) by administering an online Discrete Choice Experiment (DCE) survey.
3. To develop a framework or guideline for policy decision-making related to the efficiency and equity of contact tracing deployment

Objective of Interview:

1. To understand what attributes (factor) and levels are important to make decision for conducting COVID-19 CT

Part 1: Understanding CT for COVID-19 in Thailand

This section discusses the experience of interviewee in terms of working contact tracing in the past and/or present.

- - - - How you came to be involved in CT? are you involved in CT for COVID-19?
      - Could you briefly explain how CT is conducted for COVID-19 in Thailand?
      - What do you think are the facilitating factors for conducting CT?
      - What do you think are the barriers for conducting CT? Why?

How did you overcome these barriers?

Part 1: Proposed attributes and levels

| No | Attribute | Levels | Included in DCE (yes/no) |
| --- | --- | --- | --- |
| 1 | Contacts traced per tracer per day | 0 – 5  5 – 10  >10 |  |
| 2 | Completeness | Trace close contacts  Trace all contacts |  |
| 3 | Timeliness | Trace contacts < 24 hours  Trace contacts 24 – 48 hours  Trace contacts > 48 hours |  |
| 4 | Setting | Trace urban as priority  Trace rural as priority  Trace all (settings) |  |
| 5 | Social Economic Status (SES) | Target low SES as priority  Trace all |  |
| 6 | Incentive (to tracer) per day | THB 0/USD 0, THB 30/USD 1, THB 150/USD 5, THB 300/USD 10, THB 600/USD 20 |  |
| 7 | Epidemiology | Target sporadic case contacts  Target cluster contacts  Target community contacts |  |
| 8 | Acceptability | Contacts engage with CT voluntarily  Contacts engage with CT only if CT mandatory |  |
| 9 | Accuracy (of tests) | Test accurate  5% FP rate  10% FP rate |  |
| 10 | Privacy | Mandatory reporting of contact to authorities  Reporting of contact to authorities only with consent  Reporting of contact to authorities voluntarily |  |

DCE=Discrete choice experiment

Note: Monetary values specified in Thai baht (THB) and presented alongside US Dollar equivalent (1 USD = THB 30).

Part 2: Proposed attributes and levels

| No | Attribute | Levels | Definition |
| --- | --- | --- | --- |
| 1 | Contacts traced per tracer per day | 0 – 5  5 – 10  >10 | Required number of contacts traced per day |
| 2 | Completeness | Trace close contacts  Trace all contacts | Type of contact which may be prioritized (by risk) |
| 3 | Timeliness | Trace contacts < 24 hours  Trace contacts 24 – 48 hours  Trace contacts > 48 hours | Time to complete contact investigation after receiving notification of infection (does not include follow-up) |
| 4 | Setting | Trace urban as priority  Trace rural as priority  Trace all (settings) | Type of setting which may be prioritized to trace contact |
| 5 | Social Economic Status (SES) | Target low SES as priority  Trace all | Type of SES of contacts which may be prioritized to trace |
| 6 | Incentives (to tracer) per day | $0, $1, $5, $10, $20 / ฿0, ฿31, ฿155, ฿311, ฿623 | Contact tracers: financial incentives to be received for tracing contacts per day.  Policy makers: financial incentives which may be given to contact tracers per day |
| 7 | Epidemiology | Target sporadic case contacts  Target cluster contacts  Target community contacts | Characteristic of transmission which may be prioritized to trace contact |
| 8 | Acceptability | Contacts engage with CT voluntarily.  Contacts engage with CT only if CT mandatory | Acceptance of contacts regarding sharing information/ engaging with tracer |
| 9 | Accuracy (of tests) | Test accurate  5% False Positive rate  10% False Positive rate | Preferred accuracy level of the test to diagnose COVID-19 |
| 10 | Privacy | Mandatory reporting of contact to authorities  Reporting of contact to authorities only with consent  Reporting of contact to authorities voluntarily | Preferred policy about contacts reporting on movement to authorities |

- What attributes are important for designing contact tracing policy? why?
- Are the levels proposed appropriate?
- For the attributes which are not selected as being important, can you explain why?

Do you have any additional suggestion for other attributes and levels?

Part 3: Attributes Rank

Rank in order the importance of the following attributes for the contact tracing programme (10 = lowest importance and 1 highest importance); for example, if you consider the setting to be most important then you would assign this a rank of 1.

| No | Attribute | Levels | Rank |
| --- | --- | --- | --- |
| 1 | Contacts traced per tracer per day | 0 – 5  5 – 10  >10 |  |
| 2 | Completeness | Trace close contacts  Trace all contacts |  |
| 3 | Timeliness | Trace contacts < 24 hours  Trace contacts 24 – 48 hours  Trace contacts > 48 hours |  |
| 4 | Setting | Trace urban as priority  Trace rural as priority  Trace all (settings) |  |
| 5 | Social Economic Status (SES) | Target low SES as priority  Trace all |  |
| 6 | Incentive (to tracer) per day | $0, $1, $5, $10, $20 / ฿0, ฿31, ฿155, ฿311, ฿623 |  |
| 7 | Epidemiology | Target sporadic case contacts  Target cluster contacts  Target community contacts |  |
| 8 | Acceptability | Contacts engage with CT voluntarily  Contacts engage with CT only if CT mandatory |  |
| 9 | Accuracy (of tests) | Test accurate  5% FP rate  10% FP rate |  |
| 10 | Privacy | Mandatory reporting of contact to authorities  Reporting of contact to authorities only with consent  Reporting of contact to authorities voluntarily |  |

Part 4: Discussion on specific attributes and closing questions.

Clarification on attributes

- Equity
- Do you think there are equity issues related to contact tracing process? If yes, do you have any suggestions on how to measure these in the context of contact tracing?
- What does a “vulnerable population” mean to you in the context of contact tracing? Could you name these populations?
- Do you think that vulnerable populations need to be prioritized in the contact tracing process? Why?
- Do you think that “local access to health care” is associated with the contact tracing process? How?
- What do you think about “local access to health care” in Thailand?
- Do you think the attribute for accuracy should focus on the result of the test? How would you define accuracy of the contact tracing process instead of the test itself?

Closing questions

- What does a successful contact tracing policy mean to you?
- Do you have any additional comments?

## S4. Main survey questionnaire

**What is this research about?**

This study aims to elicit the value accorded to equity versus efficiency by representative groups of contact tracing (CT) stakeholders. The results of the study can be used to raise awareness on the lack of equity-specific key performance indicators (KPIs). Later, findings can be used for modelling CT deployment options that maximize social welfare allowing for inequity, moving thus from purely cost-effectiveness considerations that are likely to perpetuate inequities.

We would like to learn about contact tracers’ thoughts on the different aspects of contact tracing for COVID-19. Also, we are interested in your views on whether the introduction of financial rewards would be acceptable to contact tracer and policy makers.

Part 1: Survey eligibility

1. Are you 21 years of age or older?

- Yes
- No

1. Have you ever been involved directly or indirectly in the work of contact tracing for COVID-19?

- Yes
- No

[If response is NO to 1 or 2 then NOT eligible to participate.] Thank you for considering taking part in this survey. Based on the responses you provided you are not eligible to participate.

[If response is YES to 1 or 2 then eligible to participate.] We invited you to take part in a survey about preferences for COVID-19 contact tracing because you are 21 years or older and have had experience in contact tracing.

Part 2: Understanding attributes and levels for contact tracing

In this survey, we have described the performance of contact tracing based on 6 attributes (completeness, timeliness, cooperation, privacy, vulnerability and number of contacts), based on a review of the scientific literature and interviews with key stakeholders. The definition of each attribute is described below:

| **Attribute** | **Definition** |
| --- | --- |
| Completeness | Contact identification wherein either all contacts are targeted, or contacts are prioritized based on certain criteria |
| Timeliness | Time to reach contacts of index case (does not include follow-up) |
| Cooperation | Compliance with request of sharing information/ engaging with contact trace |
| Privacy | Who has access to the contact's personal data and how these are used |
| Vulnerability | The risk of having more severe symptoms due to COVID-19 infection  Vulnerable population can include elderly and people with chronic diseases who are not vaccinated.  In this survey, vulnerability is used as a measure of equity. |
| Number of contacts | The number of contacts (as per local definition) that an index case comes in contact with |

Part 3: Discrete Choice Experiment (DCE)

In this Section, we would like to present you with hypothetical (“make believe”) scenarios where you need to choose between two types of contact tracing programmes. There is no right or wrong answer; you may choose the one that you prefer more.

Please consider the current COVID-19 situation where you are residing when answering this section.

How would you categorise its current COVID-19 situation?

- Cluster case
- Sporadic case
- Community transmission

Suppose that these are two policy options for contact tracing, which one do you prefer? *

|  | Policy 1 | Policy 2 |
| --- | --- | --- |
| Completeness | Trace close contacts | Trace all contacts |
| Timeliness | Trace contacts within 24 hours | Trace contacts 24-48 hours |
| Cooperation | Mandatory cooperation of contacts | Voluntary cooperation of contacts |
| Privacy | Contact tracing data used for any purpose deemed suitable by government including linking to other datasets | Contact tracing data only used for contact tracing purposes |
| Vulnerability | Vulnerable contacts traced first | Equal priority given to every contact regardless of vulnerability |
| Number of contacts | Contacts of cases given equal priority regardless of number per case | Contacts of cases with higher number per case traced first |

*One of example of DCE choice sets (please see Supplementary S5 for full DCE questions)

Will you choose the option that you have selected above in real life?

- Yes
- No

Part 4: About you

Please tell us a little bit about yourself

In which country do you currently reside?

In which state and city do you currently reside?

- State/ province
- City

What is the highest education level you have completed? Please select one answer:

- Secondary school or below
- High school diploma or equivalent degree
- Bachelor/Masters/ PhD degree
- Other (please specify)
- Prefer not to say

What is your current role in contact tracing? Please select one answer:

- Contact tracer
- Contact tracing manager/supervisor
- Policy maker
- Academic/ expert in contact tracing
- Other (please specify)
- Prefer not to sa

Are you paid for your role in contact tracing?

- Yes
- No
- Prefer not to say

Part 5: Financial rewards

We are interested in the minimum level of a financial reward that contact tracers are willing to accept or policy makers are willing to pay, in exchange for conducting contact tracing (if this policy is to be implemented). This information will help us to determine the value at which a financial reward might increase the number of contacts traced.

How is contact tracing being paid currently in your country?

- Not applicable
- It is unpaid, voluntary role
- Each contact tracer is paid by a fixed hourly/daily wage
- Each contact tracers are paid by a fixed weekly/monthly wage
- Each contact tracers are paid by the number of contacts traced
- Other (please specify)

Please provide the actual amount of incentive range, in your country’s currency

If you feel that this question is not applicable to you, please skip to the next question.

- From:
- To:
- Country currency:
- Duration (e.g., per hour/day/week/month):

Do you think this amount should be higher or lower?

- Not applicable
- Higher
- Lower
- Stay the same

Part 6: Comments/Feedback

This is the end of the survey. Thank you for your participation.

- Do you have any comments or feedback for us?

____________________________________________________________________

## S5. Full DCE survey question

There are 10 blocks of DCE, and each block have 10 tasks. Below is an example of block 1.

Suppose that these are two policy options for contact tracing, which one do you prefer?

|  | Policy 1 | Policy 2 |
| --- | --- | --- |
| Completeness | Trace **close contacts** | Trace **all contacts** |
| Timeliness | Trace contacts **within 24 hours** | Trace contacts **24-48 hours** |
| Cooperation | **Mandatory cooperation** of contacts | **Voluntary cooperation** of contacts |
| Privacy | Contact tracing data **used for any purpose deemed suitable by government** including linking to other datasets | Contact tracing data **only used for contact tracing** purposes |
| Vulnerability | **Vulnerable contacts** traced **first** | **Equal priority** given to every contact regardless of vulnerability |
| Number of contacts | **Equal priority** given to the tracing every case regardless of number of contacts | Cases with a **higher number of contacts** traced **first** |

Will you choose the policy that you selected above in **real life**?

- Yes
- No

**There are now 9 scenarios left**

You are now on the second set of options. As you will see, you are now presented with the same attributes, but the level of the attribute may have changed for a few or all of the attributes

Suppose that these are two policy options for contact tracing, which one do you prefer?

|  | Policy 1 | Policy 2 |
| --- | --- | --- |
| Completeness | Trace **close contacts** | Trace **all contacts** |
| Timeliness | Trace contacts **within 24 hours** | Trace contacts **24-48 hours** |
| Cooperation | **Voluntary cooperation** of contacts | **Voluntary cooperation** of contacts |
| Privacy | Contact tracing data **used for any purpose deemed suitable by government** including linking to other datasets | Contact tracing data **only used for contact tracing** purposes |
| Vulnerability | **Equal priority** given to every contact regardless of vulnerability | **Equal priority** given to every contact regardless of vulnerability |
| Number of contacts | Cases with a **higher number of contacts** traced **first** | **Equal priority** given to the tracing every case regardless of number of contacts |

Will you choose the policy that you selected above in **real life**?

- Yes
- No

**There are now 8 scenarios left**

Suppose that these are two policy options for contact tracing, which one do you prefer?

|  | Policy 1 | Policy 2 |
| --- | --- | --- |
| Completeness | Trace **close contacts** | Trace **all contacts** |
| Timeliness | Trace contacts **more than 48 hours** | Trace contacts **more than 48 hours** |
| Cooperation | **Mandatory cooperation** of contacts | **Voluntary cooperation** of contacts |
| Privacy | Contact tracing data **only used for contact tracing** purposes | Contact tracing data **only used for contact tracing** purposes |
| Vulnerability | **Vulnerable contacts** traced **first** | **Vulnerable contacts** traced **first** |
| Number of contacts | Cases with a **higher number of contacts** traced **first** | **Equal priority** given to the tracing every case regardless of number of contacts |

Will you choose the policy that you selected above in **real life**?

- Yes
- No

**There are now 7 scenarios left**

Suppose that these are two policy options for contact tracing, which one do you prefer?

|  | Policy 1 | Policy 2 |
| --- | --- | --- |
| Completeness | Trace **close contacts** | Trace **all contacts** |
| Timeliness | Trace contacts **within 24 hours** | Trace contacts **24-48 hours** |
| Cooperation | **Mandatory cooperation** of contacts | **Voluntary cooperation** of contacts |
| Privacy | Contact tracing data **only used for contact tracing** purposes | Contact tracing data **used for any purpose deemed suitable by government** including linking to other datasets |
| Vulnerability | **Equal priority** given to every contact regardless of vulnerability | **Vulnerable contacts** traced **first** |
| Number of contacts | Cases with a **higher number of contacts** traced **first** | **Equal priority** given to the tracing every case regardless of number of contacts |

Will you choose the policy that you selected above in **real life**?

- Yes
- No

**There are now 6 scenarios left**

Suppose that these are two policy options for contact tracing, which one do you prefer?

|  | Policy 1 | Policy 2 |
| --- | --- | --- |
| Completeness | Trace **close contacts** | Trace **all contacts** |
| Timeliness | Trace contacts **24-48 hours** | Trace contacts **more than** **48 hours** |
| Cooperation | **Voluntary cooperation** of contacts | **Mandatory cooperation** of contacts |
| Privacy | Contact tracing data **used for any purpose deemed suitable by government** including linking to other datasets | Contact tracing data **used for any purpose deemed suitable by government** including linking to other datasets |
| Vulnerability | **Vulnerable contacts** traced **first** | **Equal priority** given to every contact regardless of vulnerability |
| Number of contacts | **Equal priority** given to the tracing every case regardless of number of contacts | **Equal priority** given to the tracing every case regardless of number of contacts |

Will you choose the policy that you selected above in **real life**?

- Yes
- No

**There are now 5 scenarios left**

Suppose that these are two policy options for contact tracing, which one do you prefer?

|  | Policy 1 | Policy 2 |
| --- | --- | --- |
| Completeness | Trace **close contacts** | Trace **all contacts** |
| Timeliness | Trace contacts **24 hours** | Trace contacts **24 hours** |
| Cooperation | **Mandatory cooperation** of contacts | **Mandatory cooperation** of contacts |
| Privacy | Contact tracing data **only used for contact tracing** purposes | Contact tracing data **used for any purpose deemed suitable by government** including linking to other datasets |
| Vulnerability | **Equal priority** given to every contact regardless of vulnerability | **Equal priority** given to every contact regardless of vulnerability |
| Number of contacts | Cases with a **higher number of contacts** traced **first** | Cases with a **higher number of contacts** traced **first** |

Will you choose the policy that you selected above in **real life**?

- Yes
- No

**There are now 4 scenarios left**

Suppose that these are two policy options for contact tracing, which one do you prefer?

|  | Policy 1 | Policy 2 |
| --- | --- | --- |
| Completeness | Trace **all contacts** | Trace **close contacts** |
| Timeliness | Trace contacts **24-48 hours** | Trace contacts **24-48 hours** |
| Cooperation | **Voluntary cooperation** of contacts | **Voluntary cooperation** of contacts |
| Privacy | Contact tracing data **used for any purpose deemed suitable by government** including linking to other datasets | Contact tracing data **used for any purpose deemed suitable by government** including linking to other datasets |
| Vulnerability | **Vulnerable contacts** traced **first** | **Vulnerable contacts** traced **first** |
| Number of contacts | **Equal priority** given to the tracing every case regardless of number of contacts | Cases with a **higher number of contacts** traced **first** |

Will you choose the policy that you selected above in **real life**?

- Yes
- No

**There are now 3 scenarios left**

Suppose that these are two policy options for contact tracing, which one do you prefer?

|  | Policy 1 | Policy 2 |
| --- | --- | --- |
| Completeness | Trace **close contacts** | Trace **close contacts** |
| Timeliness | Trace contacts **more than 48 hours** | Trace contacts **24-48 hours** |
| Cooperation | **Mandatory cooperation** of contacts | **Mandatory cooperation** of contacts |
| Privacy | Contact tracing data **used for any purpose deemed suitable by government** including linking to other datasets | Contact tracing data **only used for contact tracing** purposes |
| Vulnerability | **Vulnerable contacts** traced **first** | **Vulnerable contacts** traced **first** |
| Number of contacts | **Equal priority** given to the tracing every case regardless of number of contacts | Cases with a **higher number of contacts** traced **first** |

Will you choose the policy that you selected above in **real life**?

- Yes
- No

**There are now 2 scenarios left**

Suppose that these are two policy options for contact tracing, which one do you prefer?

|  | Policy 1 | Policy 2 |
| --- | --- | --- |
| Completeness | Trace **all contacts** | Trace **all contacts** |
| Timeliness | Trace contacts **more than 48 hours** | Trace contacts **24-48 hours** |
| Cooperation | **Voluntary cooperation** of contacts | **Mandatory cooperation** of contacts |
| Privacy | Contact tracing data **only used for contact tracing** purposes | Contact tracing data **only used for contact tracing** purposes |
| Vulnerability | **Vulnerable contacts** traced **first** | **Equal priority** given to every contact regardless of vulnerability |
| Number of contacts | Cases with a **higher number of contacts** traced **first** | **Equal priority** given to the tracing every case regardless of number of contacts |

Will you choose the policy that you selected above in **real life**?

- Yes
- No

**There are now on the last scenario**

Suppose that these are two policy options for contact tracing, which one do you prefer?

|  | Policy 1 | Policy 2 |
| --- | --- | --- |
| Completeness | Trace **close contacts** | Trace **close contacts** |
| Timeliness | Trace contacts **24-48 hours** | Trace contacts **within 48 hours** |
| Cooperation | **Mandatory cooperation** of contacts | **Mandatory cooperation** of contacts |
| Privacy | Contact tracing data **used for any purpose deemed suitable by government** including linking to other datasets | Contact tracing data **only used for contact tracing** purposes |
| Vulnerability | **Vulnerable contacts** traced **first** | **Equal priority** given to every contact regardless of vulnerability |
| Number of contacts | Cases with a **higher number of contacts** traced **first** | **Equal priority** given to the tracing every case regardless of number of contacts |

Will you choose the policy that you selected above in **real life**?

- Yes
- No

## S6. LinkedIn campaign strategy

Our target audience included people with English as their profile language located in Sri Lanka, Maldives, Singapore, Indonesia, India, Bhutan, Myanmar, Nepal, Thailand, and Bangladesh (recent or permanent), and who meet any of the following attributes:

1. Job titles (current): Public health analyst, public health advisor, public health specialist, health manager, health specialist, contact tracer, healthcare manager, epidemiologist, healthcare specialist, healthcare analyst
2. Job function: Healthcare services
3. Company (current jobs): Ministry of Health (Singapore), Ministry of Health of the Republic of Indonesia, Climate Change and Health Promotion Unit, Ministry of Health and Family Welfare, Govt. of Bangladesh, Ministry of Health – Sri Lanka, Ministry of Health and Family Welfare, Government of India
4. Company industries: Hospital and Health Care
5. Member age: 18 to >55 years old
6. Member interest: Health
7. Member groups: Epidemiology and Healthcare Epidemiology, Public Health Professionals

## S6. Supplementary tables

**Table S6.1 Response by country (The relative importance of vulnerability and efficiency in COVID-19 contact tracing programmes: a discrete choice experiment; Global; 2021)**

| **Country** | **Study sample (N=181)** | |
| --- | --- | --- |
|  | **N** | **%** |
| Argentina | 1 | 0.56 |
| Australia | 2 | 1.11 |
| Austria | 1 | 0.56 |
| Bangladesh | 14 | 7.78 |
| Belize | 1 | 0.56 |
| Democratic Republic of the Congo | 1 | 0.56 |
| Ethiopia | 2 | 1.11 |
| France | 1 | 0.56 |
| Ghana | 1 | 0.56 |
| India | 44 | 24.44 |
| Indonesia | 1 | 0.56 |
| Japan | 1 | 0.56 |
| Kenya | 1 | 0.56 |
| Lao People's Democratic Republic | 1 | 0.56 |
| Malaysia | 1 | 0.56 |
| Mexico | 1 | 0.56 |
| Nepal | 30 | 16.67 |
| New Zealand | 1 | 0.56 |
| Nigeria | 9 | 5.00 |
| Pakistan | 2 | 1.11 |
| Philippines | 4 | 2.22 |
| Portugal | 2 | 1.11 |
| Sierra Leone | 1 | 0.56 |
| Singapore | 3 | 1.67 |
| Slovakia | 1 | 0.56 |
| Somalia | 1 | 0.56 |
| South Africa | 3 | 1.67 |
| Spain | 1 | 0.56 |
| Sri Lanka | 1 | 0.56 |
| Switzerland | 2 | 1.11 |
| Thailand | 36 | 20.00 |
| Ukraine | 1 | 0.56 |
| United Kingdom of Great Britain and Nothern Ireland | 1 | 0.56 |
| United Republic of Tanzania | 2 | 1.11 |
| Unites States of America | 4 | 2.22 |
| Viet Nam | 1 | 0.56 |
| Zambia | 1 | 0.56 |

**Table S6.2. Incentive’s payment mode (The relative importance of vulnerability and efficiency in COVID-19 contact tracing programmes: a discrete choice experiment; Global; 2021)**

| **Payment mode** | **Study sample (N=146)** | |
| --- | --- | --- |
|  | **N** | **%** |
| Contact tracers are paid by the level of risk of contacts traced | 4 | 2.74 |
| Contact tracers are paid by a fixed hourly/daily wage | 19 | 13.01 |
| Contact tracers are paid by a fixed weekly/monthly wage | 46 | 31.51 |
| Contact tracers are paid by the number of contacts traced | 21 | 14.38 |
| It is an unpaid, voluntary role, or required as additional duty under the current job | 50 | 34.25 |
| On contract (details of the contract not explained) | 6 | 4.11 |

**Table S6.3. Results from latent class analysis (The relative importance of vulnerability and efficiency in COVID-19 contact tracing programmes: a discrete choice experiment; Global; 2021)**

| **Attributes and levels** | **Class 1** | | **Class 2** | |
| --- | --- | --- | --- | --- |
|  | coef | p-value | coef | p-value |
| None option | 0.58 | 0.040 | -3.04 | <0.001 |
| Completeness |  |  |  |  |
| Trace close contacts | 0.02 | 0.898 | 0.17 | 0.016 |
| Trace all contacts* | - | - | - | - |
| Timeliness |  |  |  |  |
| Less than 24 hours | 0.81 | <0.001 | 0.46 | <0.001 |
| 24 to 48 hours | 0.80 | <0.001 | 0.39 | <0.001 |
| > 48 hours* | - | - | - | - |
| Cooperation |  |  |  |  |
| Mandatory cooperation of contacts | -0.07 | 0.600 | 0.21 | 0.002 |
| Voluntary cooperation of contacts |  |  |  |  |
| Privacy |  |  |  |  |
| Contact tracing data only used for contact tracing purpose | 0.09 | 0.514 | 0.05 | 0.441 |
| Contact tracing data used for any purpose deemed suitable by government including linking to other database | - | - | - | - |
| Vulnerability |  |  |  |  |
| Trace vulnerable population first | 0.19 | 0.149 | 0.22 | 0.001 |
| Equal priority given to every person regardless vulnerability | - | - | - | - |
| Number of contacts |  |  |  |  |
| Trace index case with high number of contacts first | 0.14 | 0.285 | 0.18 | 0.006 |
| Equal priority given to every person regardless of number of contacts | - | - | - | - |
| Class membership |  |  |  |  |
| Class 2: Constant |  |  | 1.56 | <0.001 |
| Class 2: Community case |  |  | -0.49 | <0.001 |
| Class 2: High- and upper-middle-income |  |  | 0.53 | <0.001 |
| Model fit |  |  |  |  |
| AIC | 3346.1 |  |  |  |
| BIC | 3476.2 |  |  |  |

*Reference

Abbreviation: AIC, Akaike information criterion; BIC, Bayesian information criterion; Coef, Coefficient.

**Table S6.4. Importance of attributes from mixed logit model The relative importance of vulnerability and efficiency in COVID-19 contact tracing programmes: a discrete choice experiment; Global; 2021)**

| ​  **​Attributes**  **​** | **Adjusted** | | | | | | | |  |
| --- | --- | --- | --- | --- | --- | --- | --- | --- | --- |
|  |  |  |  |  |  |  |  |  |  |
|  | Sporadic or cluster,​ Low middle or low income​ | | Sporadic or cluster,​ High or upper middle income​ | | Community,​ Low middle or low income​ | | Community,​  High or upper middle income​ | |  |
|  |  |  |  |  |  |  |  |  |  |
|  | Difference​ | Rank​ | Difference​ | Rank​ | Difference​ | Rank​ | Difference​ | Rank​ |  |
|  |  |  |  |  |  |  |  |  |  |
| Completeness​ | 0.10​ | 4​ | 0.10​ | 5​ | 0.29​ | 3​ | 0.29​ | 4​ |  |
| Timeliness​ | 0.71​ | 1​ | 0.78​ | 1​ | 0.71​ | 1​ | 0.78​ | 1​ |  |
| Cooperation​ | 0.20​ | 3​ | 0.20​ | 4​ | 0.20​ | 4​ | 0.20​ | 5​ |  |
| Privacy​ | 0.09​ | 5​ | 0.09​ | 6​ | 0.09​ | 5​ | 0.09​ | 6​ |  |
| Vulnerability ​ | 0.30​ | 2​ | 0.30​ | 3​ | 0.30​ | 2​ | 0.30​ | 3​ |  |
| Number of contacts​ | 0.07​ | 6​ | 0.44​ | 2​ | 0.07​ | 6​ | 0.44​ | 2​ |  |

**Table S6.5 Importance of attributes from latent class model (The relative importance of vulnerability and efficiency in COVID-19 contact tracing programmes: a discrete choice experiment: a discrete choice experiment; Global; 2021)**

| Attributes | Class 1 | | Class 2 | |
| --- | --- | --- | --- | --- |
|  | Difference | Rank | Difference | Rank |
| Completeness | 0.02 | 5 | 0.17 | 5 |
| Timeliness | 0.81 | 1 | 0.46 | 1 |
| Cooperation | -0.07 | 6 | 0.21 | 3 |
| Privacy | 0.09 | 4 | 0.05 | 6 |
| Vulnerability | 0.19 | 2 | 0.22 | 2 |
| Number of contacts | 0.14 | 3 | 0.18 | 4 |

**Table S6.6 Most preferred and least preferred policies from latent class model (The relative importance of vulnerability and efficiency in COVID-19 contact tracing programmes: a discrete choice experiment; Global; 2021)**

| Attributes | Class 1 | | Class 2 | |
| --- | --- | --- | --- | --- |
|  | MP | LP | MP | LP |
| Completeness | Trace close contacts | Trace all contacts | Trace close contacts | Trace all contacts |
| Timeliness | Less than 24 hours | 24 to 48 hours | Less than 24 hours | 24 to 48 hours |
| Cooperation | Mandatory cooperation* | Voluntary cooperation | Mandatory cooperation | Voluntary cooperation |
| Privacy | Contact tracing data only used for contact tracing purpose* |  | Contact tracing data only used for contact tracing purpose* |  |
| Vulnerability | Trace vulnerable first* | Equal priority given to every person regardless vulnerability | Trace vulnerable first | Equal priority given to every person regardless vulnerability |
| Number of contacts | Contacts of cases with higher number per case traced first* | Contacts of cases given equal priority regardless of number per case | Contacts of cases with higher number per case traced first* | Contacts of cases given equal priority regardless of number per case |

Abbreviations: LP, least preferred; MP, most preferred.

*Statistically not significant (p-value >0.05)

## S7. Summary of literature review

This literature review aims to identify potential attributes and levels for a Discrete Choice Experiment (DCE) in contact tracing (CT), focusing on the WHO SEARO region. Three online databases: PubMed, medRxiv, bioRxiv were searched for potentially relevant studies (up to 8 November 2020), and the literature search was also complemented by manual Google search.

Overall, existing literature on CT mainly focus on mathematic modelling and digital app developments, which are not within the scope of this study. Two approaches were therefore used for this review: first, literature search was performed to find DCE studies which have focused on CT; second, literature search was also performed to find concerns of CT among LMICs. The key findings are summarised on pages 1 to 4.

1. Attributes from DCEs focusing on CT

Only three DCE studies focusing on CT were found. All focused on the predicted uptake of COVID-19 contact tracing apps in Western populations (the Netherlands, the UK, and the US), therefore their relevance to this study is in doubt. Nevertheless, for the comprehensiveness of this review, the attributes and levels in these studies are summarised in the below Table 1. Description of each study can be found in Table 2.

**Table S7.1. Attributes and levels in included study (The relative importance of vulnerability and efficiency in COVID-19 contact tracing programmes: a discrete choice experiment: a discrete choice experiment; Global; 2021)**

| Jonker et al, 2020 (the Netherlands) | Frimpong & Helleringer, 2020 (the US) | Wiertz et al, 2020 (the UK) |
| --- | --- | --- |
| - - Group size (“App users can attend…”)  1. Activities up to 3 people 2. Activities up to 10 people 3. Activities up to 30 people 4. Activities up to 100 people | - - Accuracy: false notifications  1. 1 in 100 notifications received from the app is an error 2. 5 in 100 notifications received from the app is an error 3. 15 in 100 notifications received from the app is an error | - - Responsibility and oversight  1. The NHS 2. An independent oversight body 3. The government 4. A large tech company |
| - - Warning type  1. That you were close to a person who was infected in the last 2 weeks 2. At which date and time you were close to a person who was infected | - - Accuracy: sensitivity  1. You are notified about 60% of your contacts with infected app users 2. You are notified about 80% of your contacts with infected app users 3. You are notified about 95% of your contacts with infected app users | - - Additional benefits of app use  1. Priority to get tested for COVID-19 when in self-isolation 2. Priority to book food delivery slots when in self-isolation 3. Information about how busy local shops and parks are 4. No additional benefits |
| - - Who is warned  1. Only you 2. You and the local health authorities (GGD), but only with your consent 3. You and automatically the local health authorities (GGD) | - - Privacy: user details  1. App does not ask for user details (health dept. cannot contact you) 2. App asks for phone number or email (health dept.can contact you) | - - Use of app in monitoring and/or enforcing of self-isolation  1. Used to monitor self-isolation by sending alerts 2. Not used for these purposes 3. Used to enforce self-isolation with punitive means |
| - - Testing after a warning  1. Only when someone has symptoms 2. Everyone will be tested | - - Privacy: location  1. App does not collect any location data 2. App asks user for zip code 3. App tracks location (by GPS) | - - Linkage of freedom of movement to app use  1. Entirely optional (no link to freedom of movement) 2. Required for any movement 3. Required to return to work |
| - - Who can upload test results  1. Only you 2. This is done automatically by the government and/or local health authorities (GGD) | - - Privacy: data sharing  1. You make your own COVID status available only to other app users 2. You make your own COVID status available to health department 3. You make your own COVID status and list of contacts available to health department | - - Reporting of COVID-19 test result  1. Voluntary 2. Compulsory |
| - - Financial incentive (per month)  1. €0 2. €5 3. €10 | - - Price/incentive to download  1. User pays $4.99 2. App is free 3. User gets $10 4. User gets $50 5. User gets $100 | - - Length of data storage  1. Only until the COVID-19 crisis is over 2. Only for as long as necessary 3. Only for the necessary 14 days |
|  |  | - - Anonymity  1. Anonymous contact tracing 2. Identifiable contact tracing |
|  |  | - - Whether the app shares location data  1. Yes 2. No |
|  |  | - - Infection alerts  1. Alerts for confirmed and suspected cases 2. Alerts for confirmed cases |
|  |  | - - If the app can be used in other countries  1. Yes 2. No |

2. Concerns of CTs from LMICs

In total, four studies from WHO SEARO countries were found. Two studies were conducted in Bangladesh, and highlighted public mistrust of government, existing weakness in health system, corruption, and lack of regulations as potential barriers to CT (Joarder, Khaled & Zaman, 2020); a participatory syndromic surveillance was suggested as a potential local CT approach (Mahmud et al, 2020). One study was conducted in India, with risk stratification being utilised in hospital-based CT during COVID-19 (Sahoo et al, 2020). Lastly, a commentary from the Philippines stressed the importance of considering intra-country resource disparities, as well as other competing health issues that require attention (Lau et al, 2020).

Due to the scarcity of relevant studies from WHO SEARO region, the literature search was broadened to include other LMICS. 12 more studies were found. Six studies focused on CT experiences with Ebola among African countries, while CT experiences with Zika, H1N1, MERS, as well as hypothetical scenarios including scabies, shigella and mumps were also included. For clarity, the key facilitators, barriers to and concerns of CT of the included studies are summarised in Figures 1 and 2. Description of each study can be found in Table 3.

In terms of equity, Glover et al (2020) and Shadmi et al (2020) echoed Lau et al’s comments that intra-country disparities are important. Glover et al developed a conceptual framework for identifying equity harms due to COVID-19 policies, with disparities in areas such as place of residence, race, ethnicity, culture, language, occupation, gender/sex, religion, education, socioeconomic status, social capital, age and disability.

**Figure S7.1. Facilitators to CT (Megnin-Viggars et al, 2020) (The relative importance of vulnerability and efficiency in COVID-19 contact tracing programmes: a discrete choice experiment; Global; 2021)**


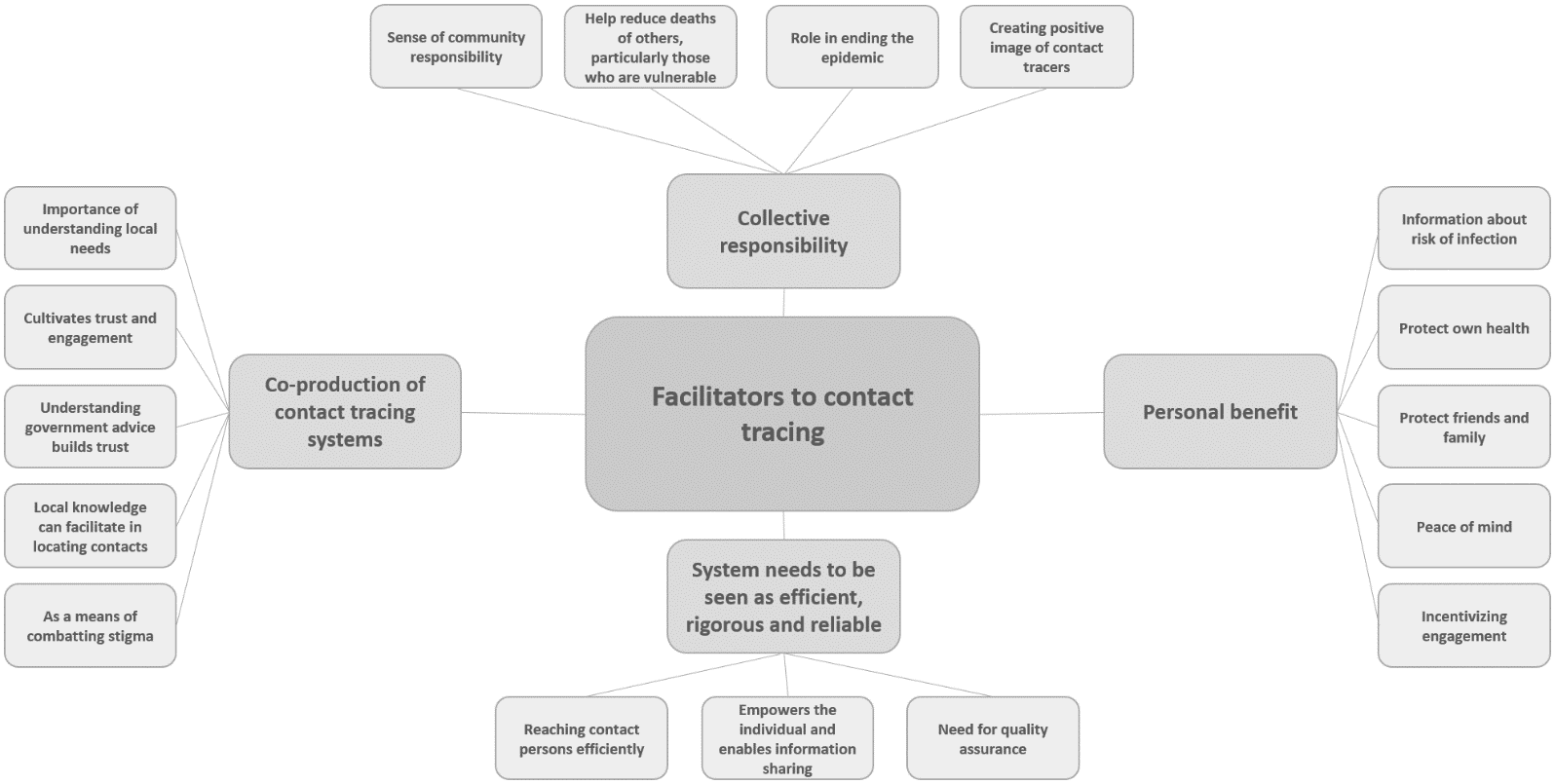
Note: The rapid review was carried out in order to gain a better understanding of the context of contact tracing. The facilitating factors mentioned above were considered when developing attributes for DCE design.

**Figure S7.2. Barriers to/concerns of CT (adapted from Megnin-Viggars et al, 2020) (The relative importance of vulnerability and efficiency in COVID-19 contact tracing programmes: a discrete choice experiment; Global; 2021)**


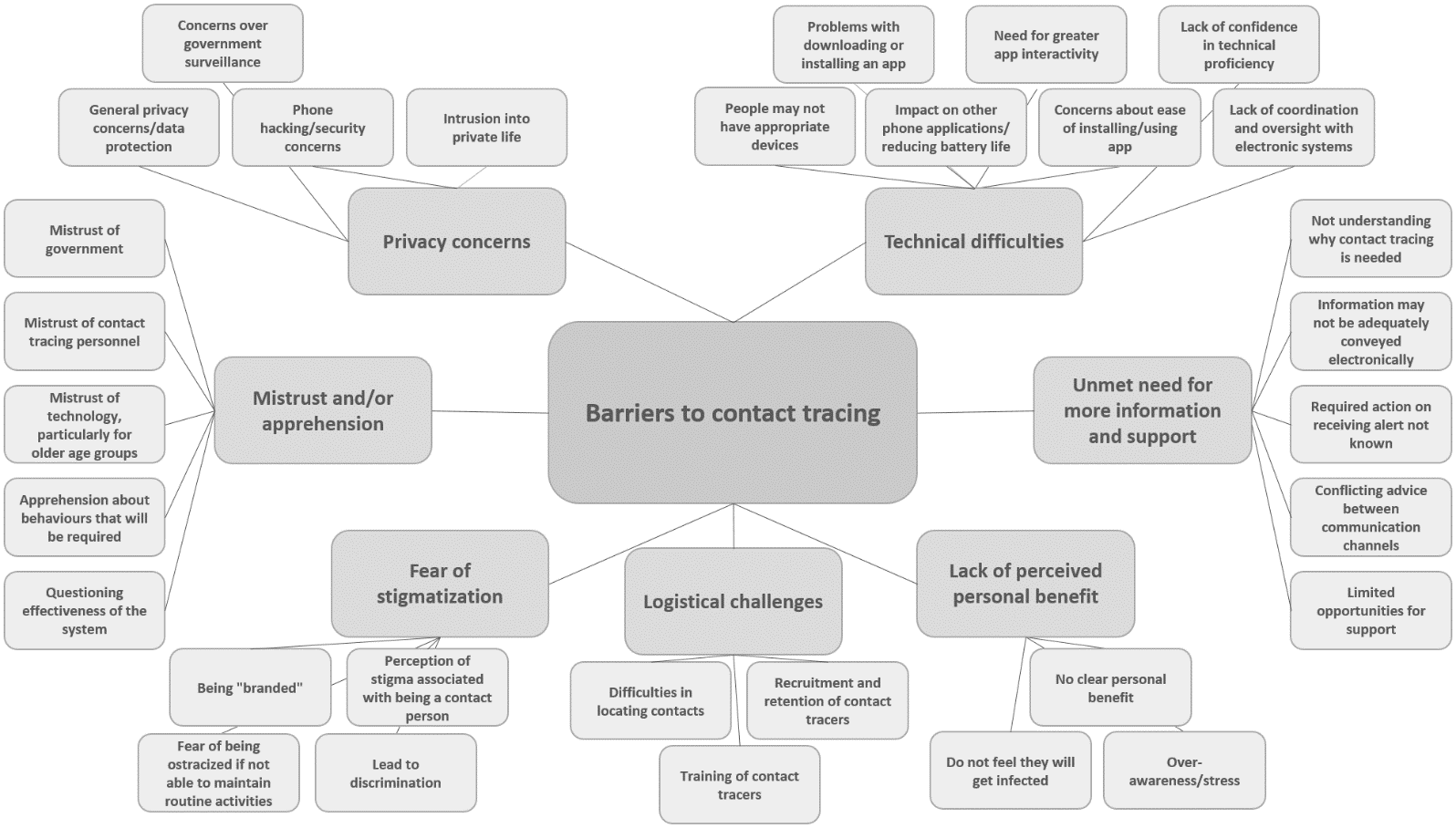


**Barriers to/Concerns of contact tracing**

**Weakness in health system**

**Enormous caseload**

**Within-country resource disparities**

**Poor area accessibility**

**Communication not culturally sensitive**

**Larger contextual factors**

**Mistrust of media**

**Mistrust of tech companies**

**Conflicts/political instability**

**Competing health issues**

**Lack of equipment for contact tracers**

**Lack of locations to self-isolate**

**Lack of locations to self-isolate**

**Need for financial/social support during quarantine**

Note: The rapid review was carried out in order to gain a better understanding of the context of contact tracing. The barriers or concerns of contact tracing depicted above were considered when developing attributes for DCE design.

| **Table S7.2. A list of published DCE studies which have focused on contact tracing (The relative importance of vulnerability and efficiency in COVID-19 contact tracing programmes: a discrete choice experiment; Global; 2021)** | | | | |
| --- | --- | --- | --- | --- |
| No. | Country | Disease | Study | Attributes |
| 1 | Netherlands | COVID-19 | [COVID-19 Contact Tracing Apps: Predicted Uptake in the Netherlands Based on a Discrete Choice Experiment (Jonker et al, 2020)](https://pubmed-ncbi-nlm-nih-gov.libproxy1.nus.edu.sg/32795998/)  The primary objective of our study is to determine the potential uptake of a contact tracing app in the Dutch population, depending on the characteristics of the app. A discrete choice experiment was conducted in a nationally representative sample of 900 Dutch respondents. Simulated maximum likelihood methods were used to estimate population average and individual-level preferences using a mixed logit model specification. The predicted app adoption rates ranged from 59.3% to 65.7% for the worst and best possible contact tracing app, respectively. The most realistic contact tracing app had a predicted adoption of 64.1%. The predicted adoption rates strongly varied by age group. Educational attainment, the presence of serious underlying health conditions, and the respondents’ stance on COVID-19 infection risks were also correlated with the predicted adoption rates but to a lesser extent. | - Group size - Warning type - Who is warned - Testing after a warning - Who can upload test results - Financial incentive (per month) |
| 2 | US | COVID-19 | [Financial Incentives for Downloading COVID–19 Digital Contact Tracing Apps (Frimpong & Helleringer, 2020)](https://www.google.com/url?sa=t&rct=j&q=&esrc=s&source=web&cd=&ved=2ahUKEwiI1vn-s_HsAhWaWX0KHdY7C8wQFjABegQIAxAC&url=https%3A%2F%2Fosf.io%2Fpreprints%2Fsocarxiv%2F9vp7x%2Fdownload&usg=AOvVaw0wHlNQ_1fmD5gJJf4xF2TO)  Contact tracing is a key approach for controlling the COVID–19 pandemic. Digital contact tracing apps have been developed to assist health departments in notifying individuals of recent exposures to SARS-CoV-2. The potential effects of digital contact tracing apps depend however on their widespread adoption. Most investigations of the determinants of adoption among potential users have focused on issues related to privacy features (e.g., who can access data, whether location is recorded) and the accuracy of the app in notifying users of exposures to SARS-CoV-2 (e.g., false notifications). In this paper, we investigate whether financial incentives might help further accelerate the adoption of digital contact tracing apps. We conducted a discrete choice experiment with an online sample of 394 US residents aged 18–69 years old. We asked participants to make a series of choices between two hypothetical versions of a digital contact tracing app characterized by several randomly selected attributes, including varying levels of financial cost or incentives to download. In this experiment, financial incentives were more than twice as important in the decision-making process about DCT app downloads than privacy and accuracy. In order to accelerate adoption, US States planning to launch digital contact tracing apps should consider offering financial incentives for download to potential users. | - Accuracy - Privacy - Price/incentives |
| 3 | UK | COVID-19 | [Predicted adoption rates of contact tracing app configurations: insights from a choice-based conjoint study with a representative sample of the UK population (Wiertz et al, 2020)](https://openaccess.city.ac.uk/id/eprint/24094/1/Whitepaper%20Contact%20Tracing%20App%20Adoption%20UK%2029%20April%202020.pdf)  Widespread adoption of a contact tracing app by the UK public is an important part of safely easing or lifting the lockdown. In this context, it is essential to understand how adoption rates are influenced by different configurations of a proposed contact tracing app. There are many implementation options that can impact app adoption. We conducted a choice-based conjoint study with a UK-wide representative sample (n=2061). Based on simulations of adoption rates for different app configurations, we recommend a contact tracing app with the following configuration: 1) oversight by NHS, 2) Priority access to testing, 3) Not used to monitor/enforce self-isolation, 4) voluntary use, 5) data stored only for 14 days, 6) voluntary reporting of test results, 7) anonymous contact tracing, 8) does not upload location data, 9) alerts for confirmed and suspected infections, 10) works internationally. This suggested configuration strikes a balance between maximising uptake of the app while protecting privacy and civil liberty. We estimate an adoption of 73.5%. | - Responsibility and oversight - Additional benefits of app use - Monitoring and/or enforcing of self-isolation - Freedom of movement - Reporting of COVID-19 test result - Length of data storage - Anonymity - Data type - Infection alerts - International use |

| **Table S7.3. A list of published studies which have described facilitators, barriers or concerns of CTs among LMICs (The relative importance of vulnerability and efficiency in COVID-19 contact tracing programmes: a discrete choice experiment; Global; 2021)** | | | | |
| --- | --- | --- | --- | --- |
| No. | Country | Disease | Study | Facilitators  /barriers or concerns |
| 1 | Bangladesh | COVID-19 | [Participatory syndromic surveillance as a tool for tracking COVID-19 in Bangladesh (Mahmud et al, 2020)](https://www.medrxiv.org/content/10.1101/2020.08.28.20183905v2.full.pdf)  Limitations in laboratory diagnostic capacity and reporting delays have hampered efforts to mitigate and control the ongoing coronavirus disease 2019 (COVID-19) pandemic globally. To augment traditional lab and hospital-based surveillance, Bangladesh established a participatory surveillance system for the public to self-report symptoms consistent with COVID-19 through multiple channels. Here, we report on the use of this system, which received over 3 million responses within two months, for tracking the COVID-19 outbreak in Bangladesh. Although we observe considerable noise in the data and initial volatility in the use of the different reporting mechanisms, the self-reported syndromic data exhibits a strong association with lab-confirmed cases at a local scale. Moreover, the syndromic data also suggests an earlier spread of the outbreak across Bangladesh than is evident from the confirmed case counts, consistent with predicted spread of the outbreak based on population mobility data. Our results highlight the usefulness of participatory syndromic surveillance for mapping disease burden generally, and particularly during the initial phases of an emerging outbreak. | - Participatory syndromic surveillance |
| 2 | Bangladesh | COVID-19 | [Health systems trust in the time of Covid-19 pandemic in Bangladesh: A qualitative exploration (Joarder, Khaled & Zaman, 2020)](https://www.medrxiv.org/content/10.1101/2020.08.05.20157768v1.full.pdf)  Lack of trust hinders care seeking, and limits community support for contact tracing, care seeking, information and communication uptake, multisectoral or multi-stakeholder engagement, and community participation. We aimed at exploring how trust might be breached and what implications this may have in COVID-19 pandemic response by the Bangladesh health systems. Methods: We conducted this qualitative research during the pandemic, through seven online focus group discussions, with purposively selected mixed-gender groups of clinicians and non-clinicians (n=50). Data were analyzed through conventional content analysis method. Results: The common thread throughout the findings was the pervasive mistrust of the people in Bangladeshi health systems in its management of COVID-19 pandemic. In addition to the existing health systems weaknesses, few others became evident throughout the progression of the pandemic, namely, the lack of coordination challenges during the preparatory phase as well as the advanced stages of the pandemic. This; compounded by the health systems and political leadership failures, lead to opportunistic corruption and lack of regulations; leading to low quality, discriminatory, or no service at all. These have trust implications, manifested in health seeking from unqualified providers, non-adherence to health advice, tension between the service seekers and providers, disapproval of the governance mechanism, misuse of already scarce resources, disinterest in community participation, and eventually loss of life and economy. Conclusions: Health sector stewards should learn the lessons from other countries, ensure multisectoral engagement involving the community and political forces, and empower the public health experts to organize and consolidate a concerted health systems effort in gaining trust in the short run, and building a resilient and responsive health system in the long. | - Public mistrust of government - Existing weakness in health system - Corruption - Lack of regulations |
| 3 | Philippines | COVID-19 | [COVID-19 response strategies: considering inequalities between and within countries (Lau et al, 2020)](https://www-ncbi-nlm-nih-gov.libproxy1.nus.edu.sg/pmc/articles/PMC7422452/pdf/12939_2020_Article_1254.pdf)  Globally, the COVID-19 pandemic has been uncharted territory, and countries and governments have faced the challenge of implementing response strategies to manage local transmission. High-income settings have the resources to devote significant resources to testing, isolation, and contact tracing. Lower-income settings are pressured to emulate such initiatives, but often lack the resources and infrastructure to do so. We highlight the impact of these between-country inequalities, the within-country inequalities, and the potential magnification of unintended consequences due to COVID-19 control measures. While there has been increasing recognition that COVID-19 response strategies need to be context-specific, discussions have primarily been focused on the national level, while differences within the country have tended to be overlooked. The global health community must be sensitive to both between and within-country inequalities as formal guidance for low-resource contexts are developed. Inappropriate copying approaches could create more unintended harm than good. | - Inter-country resource disparities - Within-country resource disparities - Other competing health issues that require attention |
| 4 | India | COVID-19 | [Hospital based contact tracing of COVID-19 patients and health care workers and risk stratification of exposed health care workers during the COVID-19 Pandemic in Eastern India (Sahoo et al, 2020)](https://www.medrxiv.org/content/10.1101/2020.11.01.20220475v1.full.pdf)  Contact tracing and subsequently quarantining of HCWs is essential to minimize further transmission. In this study, we have reported our experience of contact tracing and risk categorization in a tertiary care teaching hospital in Eastern India. This is a secondary data analysis of routine data collected from 19th March to 31st August 2020 during the process of contact tracing in a tertiary care teaching hospital in India amongst 3411 health care workers (HCWs). HCWs exposed to COVID-19 infections were categorized as per the risk stratification guidelines and the high risk cases were quarantined for 14 days and tested on 7th day of last contact. The low risk contacts were encouraged to closely monitor their symptoms and allowed to continue work. Out of 3411 contacts 890 (26.1%) were high risk contacts and 2521 (73.9%) were low risk contacts. The test positivity rate of high risk contact was 3.82% and for low risk contact was 1.9%. Number of high risk contacts was more in Non-COVID area (15.84) as compared to COVID area (0.27); p value < 0.001), amongst IPD patients (6.61) as compared to staff (4.02) and OPD (0.22) ; p value = 0.009 and when clustering of cases present (14.35) as compared to isolated cases (8.21); p value < 0.001. The case identification, risk stratification and contact tracing have helped in reducing the number of contacts with due course of time, so preventing the depletion of human resources for continuing health care. The contact tracing approach designed in the current study can also be implemented in other healthcare settings. | - Risk stratification |
| 5 | Congo | Ebola | [When is contact tracing not enough to stop an outbreak? (Dhillon & Srikrishna, 2018)](https://www-sciencedirect-com.libproxy1.nus.edu.sg/science/article/pii/S147330991830656X?via%3Dihub)  Although few studies have formally evaluated how well contact tracing works, the recent containment of the Ebola virus in the Democratic Republic of the Congo's Equateur Province, Nipah virus in India, suggest that contact tracing is effective when transmission is localised and most cases can be identified. However, there are several reasons why contact tracing might not work as effectively in situations when there are many unknown transmission chains. First, many infected individuals, much less their contacts, are not identified and contact tracing might not capture enough new infections to slow transmission. Second, contact tracing appears to have fundamental limitations in some outbreak scenarios, where some areas and contacts cannot be accessed due to insecurity created by warring factions. Similarly, during the west African epidemic, because of distrust, some communities refused to cooperate with contact tracing efforts, sometimes hiding contacts or deliberately misleading response teams. There are other instances in which contact tracing might also not be sufficiently effective. When considerable transmission is mediated by vectors that are ubiquitous and difficult to monitor or control, such as the rats that transmit Lassa fever, contact tracing can—even if executed perfectly—only keep up with the subset of transmission driven by human-to-human spread. Additionally, though contact tracing in urban areas was done effectively during smallpox eradication, the task is difficult and likely to become a more frequent challenge as the world urbanises. Third, contact tracing is operationally difficult and costly to scale up when contacts are many and spread across disparate locations. | - Abundance of unknown transmission chains - Accessibility of areas and contacts - Public distrust /refusal to cooperate - Vectors are difficult to monitor or control - Urbanisation - Operationally difficult - Costly to scale |
| 6 | West Africa | Ebola | [Role of contact tracing in containing the 2014 Ebola outbreak: a review (Saurabh & Prateek, 2017)](https://www-ncbi-nlm-nih-gov.libproxy1.nus.edu.sg/pmc/articles/PMC5636234/pdf/AFHS1701-0225.pdf)  The 2014 outbreak of Ebola virus disease which emerged in the month of March in the year 2014 in Guinea has been declared as a public health emergency of international concern. The objectives of the review article are to assess the role of contact tracing in the Ebola outbreak and to identify the challenges faced by the health workers while performing contact tracing. An extensive search of all materials related to the Ebola outbreak and contact tracing was carried out in PubMed, Medline, World Health Organization website and Google Scholar search engines. Keywords used in the search included Ebola virus disease, West-Africa, contact tracing, World Health Organization. Overall 60 articles were selected and included in the discussion.  Even though, the strategy of contact tracing has shown immense potential, but in the early part of the outbreak, it could not be implemented in most of the affected settings, due to enormous caseload and lack of health care staff. Further, identification of all possible contacts in itself is a logistical challenge as most of them cannot be traced due to the absence of any specific addresses or use of nicknames. The willingness of the contacts to get enrolled is eventually determined by their level of understanding about EVD, the associated stigma from colleagues / family members / community, and fear of being prohibited from workplace / school. Furthermore, myths like people who are listed as contacts are the ones who are more likely to die because of the disease has also interfered with the universal implementation of contact tracing. The health sector also failed miserably in establishing any sort of trust with the local communities in the affected regions. Further, factors like poor laboratory support, and the absence of an ear-marked place either to isolate or to administer treatment, never created enough support for the health workers to succeed in developing good relationships with the local community. In addition, factors like the need to promptly and comprehensively identify all the contacts, distribution of the cases / contacts in a wide geographical area, ensuring active monitoring of all contacts for three weeks, restricting movements of all the identified contacts, lack of financial assistance, poor accessibility in the affected regions due to resistance from the local residents, and extending humanitarian support services to address the non-clinical needs of contacts, also limited the utility and application of contact tracing in the local community. | - Enormous caseload - Lack of health care staff - Challenges in identifying contacts due to the absence of any specific addresses or use of nicknames - Poor health literacy /misinformation - Lack of accurate and culturally sensitive communication - Lack of trust from communities - Associated social stigma of disease - Poor laboratory support - Absence of an ear-marked place to isolate or to administer treatment - Poor accessibility in regions - Distribution of cases across large geographical area - Active monitoring of contacts |
| 7 | Sierra Leone | Ebola | [Contact Tracing during an Outbreak of Ebola Virus Disease in the Western Area Districts of Sierra Leone: Lessons for Future Ebola Outbreak Response (Olu et al, 2016)](https://www-ncbi-nlm-nih-gov.libproxy1.nus.edu.sg/pmc/articles/PMC4916168/)  Contact tracing is a critical strategy required for timely prevention and control of Ebola virus disease (EVD) outbreaks. Available evidence suggests that poor contact tracing was a driver of the EVD outbreak in West Africa, including Sierra Leone. In this article, we answered the question as to whether EVD contact tracing, as practiced in Western Area [4] districts of Sierra Leone from 2014 to 2015, was effective. The goal is to describe contact tracing and identify obstacles to its effective implementation. Mixed methods comprising secondary data analysis of the EVD case and contact tracing data sets collected from WA during the period from 2014 to 2015, key informant interviews of contact tracers and their supervisors, and a review of available reports on contact tracing were implemented to obtain data for this study. Challenges associated with effective contact tracing included lack of community trust, concealing of exposure information, political interference with recruitment of tracers, inadequate training of contact tracers, and incomplete EVD case and contact database. While the tracers noted the usefulness of community quarantine in facilitating their work, they also reported delayed or irregular supply of basic needs, such as food and water, which created resistance from the communities. Our findings confirm the results of other studies and identified incomplete identification and listing of contacts, due to a lack of disclosure of contact identification and history and inconsistent contact follow-up methods resulting in failure to reach and effectively monitor all contacts regularly, as critical challenges facing contact tracers. Inappropriate selection criteria and inadequate refresher training of contact tracers and lack of reliable contact tracing data were also identified as challenges impeding timely control of the outbreak in the WA of Sierra Leone. These findings shed more light on the dynamics of contact tracing and provide evidence that would inform more effective contact identification and listing during future outbreaks. | - Lack of community trust - Political interference - Lack of adequate training and supervision of qualified contact tracers - Lack of equipment and supplies for contact tracers - Lack of a well-managed and complete contact tracing database - Lack of provision of basic needs to quarantined contacts - Provision of wrong information about names and address - Withholding of critical information on contacts |
| 8 | Multiple countries | COVID-19,  Ebola | [Automated and partly automated contact tracing: a systematic review to inform the control of COVID-19 (Braithwaite et al, 2020)](https://www-ncbi-nlm-nih-gov.libproxy1.nus.edu.sg/pmc/articles/PMC7438082/pdf/main.pdf)  Of the 4036 studies identified, 110 full-text studies were reviewed and 15 studies were included in the final analysis and quality assessment. No empirical evidence of the effectiveness of automated contact tracing (regarding contacts identified or transmission reduction) was identified. Four of seven included modelling studies that suggested that controlling COVID-19 requires a high population uptake of automated contact-tracing apps (estimates from 56% to 95%), typically alongside other control measures. Studies of partly automated contact tracing generally reported more complete contact identification and follow-up compared with manual systems. Automated contact tracing could potentially reduce transmission with sufficient population uptake. However, concerns regarding privacy and equity should be considered. Well-designed prospective studies are needed given gaps in evidence of effectiveness, and to investigate the integration and relative effects of manual and automated systems. Large-scale manual contact tracing is therefore still key in most contexts. | - Technical issues (included poor network coverage, battery life, and quality of phones) - Successful use of app required organised flow of contact information between data managers and contact tracers - Required a concerted effort to use the app |
| 9 | Multiple countries | COVID-19, Ebola, hypothetical scenarios including scabies, shigella and mumps | [Facilitators and barriers to engagement with contact tracing during infectious disease outbreaks: A rapid review of the evidence (Megnin-Viggars et al, 2020)](https://www-ncbi-nlm-nih-gov.libproxy1.nus.edu.sg/pmc/articles/PMC7595276/pdf/pone.0241473.pdf)  Until a vaccine is developed, a test, trace and isolate strategy is the most effective method of controlling the COVID-19 outbreak. Contact tracing and case isolation are common methods for controlling infectious disease outbreaks. However, the effectiveness of any contact tracing system rests on public engagement. Numerous factors may influence an individual’s willingness to engage with a contact tracing system. Understanding these factors has become urgent during the COVID-19 pandemic. A rapid systematic review was conducted to identify papers based on primary research, written in English, and that assessed facilitators, barriers, and other factors associated with the uptake of, and engagement with, a contact tracing system. Four themes were identified as facilitators to the uptake of, and engagement with, contact tracing: collective responsibility; personal benefit; co-production of contact tracing systems; and the perception of the system as efficient, rigorous and reliable. Five themes were identified as barriers to the uptake of, and engagement with, contact tracing: privacy concerns; mistrust and/or apprehension; unmet need for more information and support; fear of stigmatization; and mode-specific challenges. | - Need for collective responsibility - Privacy concerns - Mistrust /apprehension - Unmet need for information and support - Fear of stigmatisation - Mode-specific challenges (e.g. technical difficulties) |
| 10 | Multiple countries | COVID-19 | [Effective Contact Tracing for COVID-19: A Systematic Review (Juneau et al, 2020)](https://www.medrxiv.org/content/10.1101/2020.07.23.20160234v2.full.pdf)  Contact tracing is commonly recommended to control outbreaks of COVID-19, but its effectiveness is unclear. This systematic review aimed to examine contact tracing effectiveness in the context of COVID-19. A total of 32 articles were found. All were observational or modelling studies, so the quality of the evidence was low. Observational studies (n=14) all reported that contact tracing (alone or in combination with other interventions) was associated with better control of COVID-19. Results of modelling studies (n=18) depended on their assumptions. Under assumptions of prompt and thorough tracing with no further transmission, they found that contact tracing could stop an outbreak (e.g. by reducing the reproduction number from 2.2 to 0.57) or that it could reduce infections (e.g. by 24%-71% with a mobile tracing app). Under assumptions of slower, less efficient tracing, modelling studies suggested that tracing could slow, but not stop COVID-19.  Observational and modelling studies suggest that contact tracing is associated with better control of COVID-19. Its effectiveness likely depends on a number of factors, including how many and how fast contacts are traced and quarantined, and how effective quarantines are at preventing further transmission. A cautious interpretation suggests that to stop the spread of COVID-19, public health practitioners have 2-3 days from the time a new case develops symptoms to isolate the case and quarantine at least 80% of its contacts, and that once isolated, cases and contacts should infect zero new cases. Less efficient tracing may slow, but not stop, the spread of COVID-19. Inefficient tracing (with delays of 4-5+ days or less than 60% of contacts quarantined with no further transmission) may not contribute meaningfully to control of COVID-19. | - Delays in test results - Ineffective isolation and quarantine - Need for financial and social support among those quarantined - Large caseloads - Lack of full disclosure of contacts - Costly operation - Limited adoption of CT apps |
| 11 | Multiple countries | Ebola, Zika, H1N1, MERS | [Community engagement for COVID-19 prevention and control: A Rapid Evidence Synthesis (Gilmore et al, 2020)](https://gh.bmj.com/content/bmjgh/5/10/e003188.full.pdf)  A rapid evidence review was conducted to identify how community engagement is used for infectious disease prevention and control during epidemics. Three databases were searched in addition to extensive snowballing for grey literature. Previous epidemics were limited to Ebola, Zika, SARS, Middle East respiratory syndrome and H1N1 since 2000. No restrictions were applied to study design or language. From 1112 references identified, 32 articles met our inclusion criteria, which detail 37 initiatives. Six main community engagement actors were identified: local leaders, community and faith-based organisations, community groups, health facility committees, individuals and key stakeholders. These worked on different functions: designing and planning, community entry and trust building, social and behaviour change communication, risk communication, surveillance and tracing, and logistics and administration. COVID-19’s global presence and social transmission pathways require social and community responses. This may be particularly important to reach marginalised populations and to support equity-informed responses. Aligning previous community engagement experience with current COVID-19 community-based strategy recommendations highlights how communities can play important and active roles in prevention and control. Countries worldwide are encouraged to assess existing community engagement structures and use community engagement approaches to support contextually specific, acceptable and appropriate COVID-19 prevention and control measures. | - Early engagement - Ongoing processes, reassessed and modified as needed - Multisectoral engagement - Decentralised governance - Clear roles and responsibilities for all stakeholders - Strong linkage with community-level response efforts - Use and engagement with pre-existing actors (if appropriate) - Open communication with clear, two-way channels - Lack of contextual understanding - Engagement with inappropriate actors - Lack of trust in government, media, and organisations - Inconsistent messaging - Unclear responsibilities for stakeholders - Inadequate training and/or support structures - Lack of resources or incentivisation - Weak health support infrastructure - Broader contextual factors: e.g. conflict, poverty, mobility |
| 12 | Colombia | COVID-19 | [Impact of contact tracing on COVID-19 mortality: An impact evaluation using surveillance data from Colombia (Vecino-Ortiz et al, 2020)](https://www.medrxiv.org/content/10.1101/2020.08.14.20158535v1)  This study assesses the impact of contact tracing in a middle-income country and provides data to support the expansion of contact tracing strategies with the aim of improving infection control. We obtained publicly available data on all confirmed COVID-19 cases in Colombia between March 2 and June 16, 2020. (N=54,931 cases over 135 days of observation). We proxied contact tracing performance as the proportion of cases identified through contact tracing out of all cases identified, as suggested by WHO guidelines. We calculated the daily proportion of cases identified through contact tracing across 37 geographical units. Further, we used a sequential log-log fixed-effects model to estimate the 21-days, 28-days, 42-days and 56-days lagged impact of the proportion of cases identified through contact tracing on the daily number of COVID-19 deaths. Both the proportion of cases identified through contact tracing and the daily number of COVID-19 deaths are smoothed using 7-day moving averages. We found that a 10 percent increase in the proportion of cases identified through contact tracing is related to COVID-19 mortality reductions between 0.8% and 3.4%. Our models explain between 47%-70% of the variance in mortality. Contact tracing is instrumental to contain infectious diseases and its prioritization as a surveillance strategy will have a substantial impact on reducing deaths while minimizing the impact on the fragile economic systems of lower and middle-income countries. This study provides lessons for other LMIC. | - Lack of strong public health structure - Lack of well-trained human resources - Lack of funding - Limited mobile network coverage |
| 13 | 92 LMICs | COVID-19 | [The health sector cost of different policy responses to COVID-19 in low- and middle- income countries (Torres-Rueda et al, 2020)](https://www.medrxiv.org/content/10.1101/2020.08.23.20180299v1.full.pdf)  Much attention has focussed in recent months on the impact that COVID-19 has on health sector capacity, including critical care bed capacity and resources such as personal protective equipment. However, much less attention has focussed on the overall cost to health sectors, including the full human resource costs and the health system costs to address the pandemic. Here we present estimates of the total costs of COVID-19 response in low- and middle-income countries for different scenarios of COVID-19 mitigation over a one year period. We find costs vary substantially by setting, but in some settings even mitigation scenarios place a substantial fiscal impact on the health system. For contact tracing, the mean unit cost per activity is estimated to be US$2.93 in low income countries (LICs), US$9.84 in lower-middle income countries (LMICs), and US$19.24 in upper-middle income countries (UMICs). Further quarantine of contacts would cost an additional US$2.83 in LICs, US$6.36 in LMICs, and US$15.81 in UMICs. We conclude that the choices facing many low- and middle- income countries, without further rapid emergency financial support, are stark, between fully funding an effective COVID-19 reponse or other core essential health services. | - Mean unit cost of contact tracing is US$2.93 in low income countries (LICs), US$9.84 in lower-middle income countries (LMICs) - Further quarantine of contacts would cost an additional US$2.83 in LICs, US$6.36 in LMICs |
| 14 | 79 territories | COVID-19 | [Ranking the effectiveness of worldwide COVID-19 government interventions (Haug et al, 2020)](https://www.medrxiv.org/content/10.1101/2020.07.06.20147199v2.full.pdf)  Assessing the effectiveness of Non-Pharmaceutical Interventions (NPIs) to mitigate the spread of SARS-CoV-2 is critical to inform future preparedness response plans. Here we quantify the impact of 6,068 hierarchically coded NPIs implemented in 79 territories on the effective reproduction number, Rt, of COVID-19. It is interesting to comment on the impact that "Enhancing testing capacity" and "Tracing and tracking" would have had if adopted at different points in times. Counterintuitively, tracing, tracking and testing measures should display a short-term increase of Rt if they are effective, as more cases will be found. For countries implementing these measures early this is indeed what we find. However, countries implementing these NPIs later did not necessarily find more cases, as shown by the corresponding decrease in Rt, We focused on March and April 2020, a period in which many countries had surged in positive cases that overwhelmed their testing and tracing capacities, which rendered the corresponding NPIs ineffective. | - Late implementation of contact tracing renders the intervention ineffective |
| 15 | 13 countries including Thailand and India | COVID-19 | [Health equity and COVID-19: global perspectives (Shadmi et al, 2020)](https://equityhealthj.biomedcentral.com/articles/10.1186/s12939-020-01218-z)  The COVID-19 is disproportionally affecting the poor, minorities and a broad range of vulnerable populations, due to its inequitable spread in areas of dense population and limited mitigation capacity due to high prevalence of chronic conditions or poor access to high quality public health and medical care. Moreover, the collateral effects of the pandemic due to the global economic downturn, and social isolation and movement restriction measures, are unequally affecting those in the lowest power strata of societies. To address the challenges to health equity and describe some of the approaches taken by governments and local organizations, we have compiled 13 country case studies from various regions around the world: China, Brazil, Thailand, Sub Saharan Africa, Nicaragua, Armenia, India, Guatemala, United States of America [5], Israel, Australia, Colombia, and Belgium. This compilation is by no-means representative or all inclusive, and we encourage researchers to continue advancing global knowledge on COVID-19 health equity related issues, through rigorous research and generation of a strong evidence base of new empirical studies in this field. | - Universal health coverage /medical insurance policies improved access to treatment (equity) - Special care services for disabled people so they can access health services (equity) - Existing primary healthcare network and volunteers help with CT - CT and quarantine in dedicated facilities provided for free for citizens - Intra-country regional inequalities - Racial inequality, and other vulnerable populations (e.g. indigenous, prisoners, homeless) |
| 16 | Multiple countries | COVID-19 | [A framework for identifying and mitigating the equity harms of COVID-19 policy interventions (Glover et al, 2020)](https://www.ncbi.nlm.nih.gov/pmc/articles/PMC7280094/)  Coronavirus disease 2019 (COVID-19) is a global pandemic. Governments have implemented combinations of “lockdown” measures of various stringencies, including school and workplace closures, cancellations of public events, and restrictions on internal and external movements. These policy interventions are an attempt to shield high-risk individuals and to prevent overwhelming countries' healthcare systems, or, colloquially, “flatten the curve.” However, these policy interventions may come with physical and psychological health harms, group and social harms, and opportunity costs. These policies may particularly affect vulnerable populations and not only exacerbate pre-existing inequities but also generate new ones. We developed a conceptual framework to identify and categorize adverse effects of COVID-19 lockdown measures. We based our framework on Lorenc and Oliver's framework for the adverse effects of public health interventions and the PROGRESS-Plus equity framework. To test its application, we purposively sampled COVID-19 policy examples from around the world and evaluated them for the potential physical, psychological, and social harms, as well as opportunity costs, in each of the PROGRESS-Plus equity domains: Place of residence, Race/ethnicity, Occupation, Gender/sex, Religion, Education, Socioeconomic status, Social capital, Plus (age, and disability).  We found examples of inequitably distributed adverse effects for each COVID-19 lockdown policy example, stratified by a low- or middle-income country and high-income country, in every PROGRESS-Plus equity domain. We identified the known policy interventions intended to mitigate some of these adverse effects. The same harms (anxiety, depression, food insecurity, loneliness, stigma, violence) appear to be repeated across many groups and are exacerbated by several COVID-19 policy interventions. Our conceptual framework highlights the fact that COVID-19 policy interventions can generate or exacerbate interactive and multiplicative equity harms. Applying this framework can help in three ways: (1) identifying the areas where a policy intervention may generate inequitable adverse effects; (2) mitigating the policy and practice interventions by facilitating the systematic examination of relevant evidence; and (3) planning for lifting COVID-19 lockdowns and policy interventions around the world. | - To identify equity harms due to COVID-19 policies, consider: place of residence, race, ethnicity, culture, language, occupation, gender/sex, religion, education, socioeconomic status, social capital, age, disability - Harms can include physical health, psychological health, harms to certain groups or parts of society, and opportunity cost |

| **Table S7.4. A list of published studies which have described facilitators, barriers or concerns of Contact tracing in HICs or focus on CT apps (The relative importance of equity and efficiency in COVID-19 contact tracing programmes: a discrete choice experiment; Global; 2021)** | | | | |
| --- | --- | --- | --- | --- |
| No. | Country | Disease | Study | Facilitators  / barriers or concerns |
| 1 | France, Germany, Italy, UK, US | COVID-19 | [Acceptability of App-Based Contact Tracing for COVID-19: Cross-Country Survey Study (Altmann et al, 2020)](https://www-ncbi-nlm-nih-gov.libproxy1.nus.edu.sg/pmc/articles/PMC7458659/)  A largescale, multicountry study (N=5995) was conducted to measure public support for the digital contact tracing of COVID-19 infections. We ran anonymous online surveys in France, Germany, Italy, the United Kingdom, and the United States. We measured intentions to use a contact-tracing app across different installation regimes (voluntary installation vs automatic installation by mobile phone providers) and studied how these intentions vary across individuals and countries. We found strong support for the app under both regimes, in all countries, across all subgroups of the population, and irrespective of regional-level COVID-19 mortality rates. We investigated the main factors that may hinder or facilitate uptake and found that concerns about cybersecurity and privacy, together with a lack of trust in the government, are the main barriers to adoption. | - Lack of trust in government - Concerns about cybersecurity   Concerns about privacy |
| 2 | Ireland | COVID-19 | [A national survey of attitudes to COVID-19 digital contact tracing in the Republic of Ireland (O’Callaghan et al 2020)](https://www-ncbi-nlm-nih-gov.libproxy1.nus.edu.sg/pmc/articles/PMC7561439/#!po=17.8571)  A national survey was conducted among the Irish population to examine barriers and levers to the use of a contact tracing App. A total of 8088 responses were received, with all 26 counties of the Republic of Ireland represented. Fifty-four percent of respondents said they would definitely download a contact-tracing App, while 30% said they would probably download a contact tracing App. Ninety-five percent of respondents identified at least one reason for them to download such an App, with the most common reasons being the potential for the App to help family members and friends and a sense of responsibility to the wider community. Fifty-nine percent identified at least one reason not to download the App, with the most common reasons being fear that technology companies or the government might use the App technology for greater surveillance after the pandemic. Concerns raised regarding privacy and data security will be critical if the App is to achieve the large-scale adoption and ongoing use required for its effective operation. | - Fear of surveillance by the government /tech companies - Fear phone will be hacked   Data protection |
| 3 | Poland | COVID-19 | [The acceptance of Covid-19 tracking technologies: The role of perceived threat, lack of control, and ideological beliefs (Wnuk et al 2020)](https://www-ncbi-nlm-nih-gov.libproxy1.nus.edu.sg/pmc/articles/PMC7485859/)  New technological solutions play an important role in preventing the spread of Covid-19. Many countries have implemented tracking applications or other surveillance systems, which may raise concerns about privacy and civil rights violations but may be also perceived by citizens as a way to reduce threat and uncertainty. Our research examined whether feelings evoked by the pandemic (perceived threat and lack of control) as well as more stable ideological views predict the acceptance of such technologies. In two studies conducted in Poland, we found that perceived personal threat and lack of personal control were significantly positively related to the acceptance of surveillance technologies, but their predictive value was smaller than that of individual differences in authoritarianism and endorsement of liberty. Moreover, we found that the relationship between the acceptance of surveillance technologies and both perceived threat and lack of control was particularly strong among people high in authoritarianism. Our research shows that the negative feelings evoked by the unprecedented global crisis may inspire positive attitudes towards helpful but controversial surveillance technologies but that they do so to a lesser extent than ideological beliefs. | - Perceived personal threat - Lack of personal control |
| 4 | Jordan | COVID-19 | [COVID-19 Contact-Tracing Technology: Acceptability and Ethical Issues of Use (Abuhammad et al, 2020)](https://www-ncbi-nlm-nih-gov.libproxy1.nus.edu.sg/pmc/articles/PMC7509307/#__ffn_sectitle)  The purpose of this study was to determine the acceptability of COVID-19 contact-tracing technology and ethical issues of use. A cross-sectional questionnaire-based study was used. The target population was Jordanian adults (>18 years). The survey was distributed to a convenience sample of 2000 general public in Jordan. The results found that the number of people who accept to use COVID-19 contact-tracing technology was 71.6%. However, the percentage of people who were using this technology was 37.8. The main ethical concerns for many of participants were privacy, voluntariness, and beneficence of the data. Only income and living area were predictors for acceptability and use of tracing technology (p≤ 0.01). The majority of Jordanians accept the implementation of contact-tracing technology for COVID-19 infection. Among ethical concerns of the implementation of such technology were privacy, beneficence and voluntariness. | - Whether technology is regulated - objective and description of the technology - how it works - sponsors of this technology - potential burdens - possible benefits - expected required time - Incentives - Voluntariness - Data access - Data privacy   How the information will be used |
| 5 | Belgium | COVID-19 | [Adoption of a Contact Tracing App for Containing COVID-19: A Health Belief Model Approach (Walrave et al, 2020)](https://www-ncbi-nlm-nih-gov.libproxy1.nus.edu.sg/pmc/articles/PMC7470174/)  To track and reduce the spread of COVID-19, apps have been developed to identify contact with individuals infected with SARS-CoV-2 and warn those who are at risk of having contracted the virus. However, the effectiveness of these apps depends highly on their uptake by the general population. The present study investigated factors influencing app use intention, based on the health belief model. A survey was administered in Flanders, Belgium, to 1500 respondents, aged 18 to 64 years. Structural equation modeling was used to investigate relationships across the model’s constructs. In total, 48.70% (n=730) of respondents indicated that they intend to use a COVID-19 tracing app. The most important predictor was the perceived benefits of the app, followed by self-efficacy and perceived barriers. Perceived severity and perceived susceptibility were not related to app uptake intention. Moreover, cues to action (ie, individuals’ exposure to [digital] media content) were positively associated with app use intention. As the respondents’ age increased, their perceived benefits and self-efficacy for app usage decreased. Initiatives to stimulate the uptake of contact tracing apps should enhance perceived benefits and self-efficacy. A perceived barrier for some potential users is privacy concerns. Therefore, when developing and launching an app, clarification on how individuals’ privacy will be protected is needed. To sustain perceived benefits in the long run, supplementary options could be integrated to inform and assist users. | - Privacy concerns - Perceived benefits - Self-efficacy |
| 6 | Germany, Austria, Switzerland | COVID-19 | [Digital contact-tracing during the Covid-19 pandemic: an analysis of newspaper coverage in Germany, Austria, and Switzerland (Amann, Sleigh & Vayena, 2020)](https://www.medrxiv.org/content/10.1101/2020.10.22.20216788v1)  Governments around the globe have started to develop and deploy digital contact tracing apps to gain control over the spread of the novel coronavirus (Covid-19). The appropriateness and usefulness of these technologies as a containment measure have since sparked political and academic discussions globally. The present paper contributes to this debate through an exploration of how the national daily newspapers in Germany, Austria, and Switzerland reported on the development and adoption of digital contact-tracing apps during early and after stages of the lockdown. We conducted thematic analysis on news coverage published between January and May 2020 in high-circulation national daily newspapers (print) from Germany, Austria, and Switzerland. A total of 148 newspaper articles were included in the final analysis. From our analysis emerged six core themes of the development and adoption of digital contact tracing apps: 1) data governance; 2) role of IT giants; 3) scientific rigor; 4) voluntariness; 5) functional efficacy; 6) role of the app. These results shed light on the different facets of discussion regarding digital contact tracing as portrayed in German-speaking media. As news coverage can serve as a proxy for public perception, this study complements emerging survey data on public perceptions of digital contact tracing apps by identifying potential issues of public concern. | - Data governance - Role of IT giants - Scientific rigor - Voluntariness - Functional efficacy - Role of the app |
| 7 | US (modelling) | COVID-19 | [Contact tracing efficiency, transmission heterogeneity, and accelerating COVID-19 epidemics (Gardner & Kilpatrick, 2020)](https://www.medrxiv.org/content/10.1101/2020.09.04.20188631v2.full.pdf)  Manual contact tracing is a time-consuming process and as case numbers increase it takes longer to reach each cases' contacts, leading to additional virus spread. Delays between symptom onset and being tested (and receiving results), and a low fraction of symptomatic cases being tested and traced can also reduce the impact of contact tracing on transmission. We examined the relationship between cases and delays and the pathogen reproductive number Rt, and the implications for infection dynamics using a stochastic compartment model of SARS-CoV-2. We found that Rt increases sigmoidally with the number of cases due to decreasing contact tracing efficacy. This relationship results in accelerating epidemics because Rt increases, rather than declines, as infections increase. Shifting contact tracers from locations with high and low case burdens relative to capacity to locations with intermediate case burdens maximizes their impact in reducing Rt (but minimizing total infections is more complicated). Contact tracing efficacy also decreased with increasing delays between symptom onset and tracing and with lower fraction of symptomatic infections being tested. Finally, testing and tracing reductions in Rt can sometimes greatly delay epidemics due to the highly heterogeneous transmission dynamics of SARS-CoV-2. These results demonstrate the importance of having an expandable or mobile team of contact tracers that can be used to control surges in cases, and the value of easy access, high testing capacity and rapid turn-around of testing results, as well as outreach efforts to encourage symptomatic infections to be tested immediately after symptom onset. | - Expandable or mobile team of contact tracers that can be used to control surges in cases |
| 8 | US | COVID-19 | [Tracing and testing the COVID-19 contact chain: cost-benefit tradeoffs (Kim et al, 2020)](https://www.medrxiv.org/content/10.1101/2020.10.01.20205047v2.full.pdf)  Traditional contact tracing for COVID-19 tests the direct contacts of those who test positive even if the contacts do not show any symptom. But, why should the testing stop at direct contacts, and not test secondary, tertiary contacts or even contacts further down? The question arises because by the time an infected individual is tested the infection starting from him may have infected a chain of individuals. One deterrent in testing long chains of individuals right away may be that it substantially increases the testing load, or does it? We investigate the costs and benefits of testing the contact chain of an individual who tests positive. For this investigation, we utilize multiple human contact networks, spanning two real-world data sets of spatio-temporal records of human presence over certain periods of time, as also networks of a classical synthetic variety. Over the diverse set of contact patterns, we discover that testing the contact chain can both substantially reduce over time both the cumulative infection count and the testing load. We consider elements of human behavior that enhance the spread of the disease and lower the efficacy of testing strategies, and show that testing the contact chain enhances the resilience to adverse impacts of these elements. We also discover a phenomenon of diminishing return beyond a threshold value on the depth of the chain to be tested in one go, the threshold then provides the most desirable tradeoff between benefit in terms of reducing the cumulative infection count, enhancing resilience to adverse impacts of human behavior, and cost in terms of increasing the testing load. | - Public cooperativity - Privacy concerns |
| 9 | Singapore | COVID-19 | [Health equity considerations in COVID-19: geospatial network analysis of the COVID-19 outbreak in the migrant population in Singapore (Yi et al, 2020)](https://academic.oup.com/jtm/advance-article/doi/10.1093/jtm/taaa159/5902308)  Low-wage dormitory-dwelling migrant workers in Singapore were disproportionately affected by coronavirus disease 2019 (COVID-19) infection. This was attributed to communal living in high-density and unhygienic dormitory settings and a lack of inclusive protection systems. However, little is known about the roles of social and geospatial networks in COVID-19 transmission. The study examined the networks of non-work–related activities among migrant workers to inform the development of lockdown exit strategies and future pandemic preparedness. A population-based survey was conducted with 509 migrant workers across the nation, and it assessed dormitory attributes, social ties, physical and mental health status, COVID-19-related variables and mobility patterns using a grid-based network questionnaire. Mobility paths from dormitories were presented based on purposes of visit. Two-mode social networks examined the structures and positions of networks between workers and visit areas with individual attributes. COVID-19 risk exposure was associated with the density of dormitory, social ties and visit areas. The migrant worker hub in the city centre was the most frequently visited for essential services of grocery shopping and remittance, followed by south central areas mainly for social gathering. The hub was positioned as the core with the highest degree of centrality with a cluster of workers exposed to COVID-19. Social and geospatial networks of migrant workers should be considered in the implementation of lockdown exit strategies while addressing the improvement of living conditions and monitoring systems. Essential services, like remittance and grocery shopping at affordable prices, need to be provided near to dormitories to minimize excess gatherings. | - proximity to essential services (to avoid overcrowding) |

Note: These studies have been conducted in high-income countries or focus only on CT apps.

# **References**

1. The World Heatlh Organization (WHO). Critical preparedness, readiness and response actions for COVID-19 2021 [updated 27 May 2021. Available from: <https://www.who.int/publications/i/item/critical-preparedness-readiness-and-response-actions-for-covid-19>.

2. Reed Johnson F, Lancsar E, Marshall D, Kilambi V, Mühlbacher A, Regier DA, et al. Constructing experimental designs for discrete-choice experiments: report of the ISPOR Conjoint Analysis Experimental Design Good Research Practices Task Force. Value in health : the journal of the International Society for Pharmacoeconomics and Outcomes Research. 2013;16(1):3-13.

3. Sawtooth Software Inc. Technical paper series: The CBC System for Choice-Based Conjoint Analysis. 2013.

4. Fiebig DG, Keane MP, Louviere J, Wasi N. The Generalized Multinomial Logit Model: Accounting for Scale and Coefficient Heterogeneity. Marketing Science. 2010;29(3):393-421.

5. Rydén A, Chen S, Flood E, Romero B, Grandy S. Discrete Choice Experiment Attribute Selection Using a Multinational Interview Study: Treatment Features Important to Patients with Type 2 Diabetes Mellitus. The Patient - Patient-Centered Outcomes Research. 2017;10(4):475-87.
